# SARS-CoV-2 shedding dynamics and transmission in immunosuppressed patients

**DOI:** 10.1080/21505594.2022.2101198

**Published:** 2022-07-26

**Authors:** Jee-Soo Lee, Ki Wook Yun, Hyeonju Jeong, Boram Kim, Man Jin Kim, Jae Hyeon Park, Ho Seob Shin, Hyeon Sae Oh, Hobin Sung, Myung Gi Song, Sung Im Cho, So Yeon Kim, Chang Kyung Kang, Pyoeng Gyun Choe, Wan Beom Park, Nam Joong Kim, Myoung-Don Oh, Eun Hwa Choi, Seungman Park, Taek Soo Kim, Jung-Hee Lee, Heungsup Sung, Sung Sup Park, Moon-Woo Seong

**Affiliations:** a Department of Laboratory Medicine, Seoul National University Hospital, Seoul National University College of Medicine, Seoul, Republic of Korea; b Department of Pediatrics, Seoul National University College of Medicine, Seoul, Republic of Korea; c Department of Internal Medicine, Gyeonggi Provincial Medical Center, Ansung Hospital, Anseong Gyeonggi-do, Republic of Korea; d Department of Laboratory Medicine, National Medical Center, Seoul, Republic of Korea; e Department of Internal Medicine, Seoul National University College of Medicine, Seoul, Republic of Korea; f Department of Laboratory Medicine, Seegene Medical Foundation, Seoul, Republic of Korea; g Department of Haematology, Asan Medical Center, University of Ulsan College of Medicine, Seoul, Republic of Korea; h Department of Laboratory Medicine, Asan Medical Center, University of Ulsan College of Medicine, Seoul, Republic of Korea

**Keywords:** SARS-CoV-2, variant of concern, whole genome sequencing, mutation dynamics, long-term viral shedding

## Abstract

Severe acute respiratory syndrome coronavirus 2 (SARS-CoV-2) variants of concern have been emerging. However, knowledge of temporal and spatial dynamics of SARS-CoV-2 is limited. This study characterized SARS-CoV-2 evolution in immunosuppressed patients with long-term SARS-CoV-2 shedding for 73–250 days, without specific treatment. We conducted whole-genome sequencing of 27 serial samples, including 26 serial samples collected from various anatomic sites of two patients and the first positive sample from patient 2‘s mother. We analysed the intrahost temporal dynamics and genomic diversity of the viral population within different sample types. Intrahost variants emerging during infection showed diversity between individual hosts. Remarkably, N501Y, P681R, and E484K, key substitutions within spike protein, emerged *in vivo* during infection and became the dominant population. P681R, which had not yet been detected in the publicly available genome in Korea, appeared within patient 1 during infection. Mutually exclusive substitutions at residues R346 (R346S and R346I) and E484 (E484K and E484A) of spike protein and continuous turnover of these substitutions occurred. Unique genetic changes were observed in urine samples. A household transmission from patient 2 to his mother, at least 38 days after the diagnosis, was characterized. Viruses may differently mutate and adjust to the host selective pressure, which could enable the virus to replicate efficiently for fitness in each host. Intrahost variants could be candidate variants likely to spread to the population eventually. Our findings may provide new insights into the dynamics of SARS-CoV-2 in response to interactions between the virus and host.

## Introduction

The ongoing coronavirus disease 2019 (COVID-19) pandemic has reached a phase with new variants of severe acute respiratory syndrome coronavirus 2 (SARS-CoV-2) emerging, which may confer evolutionary advantages on the virus [1,2]. Recently, SARS-CoV-2 variants of concern (VOC) including alpha (B.1.1.7 characterized by Δ69/70 and N501Y), beta (B.1.351 with mutations K417N, E484K, and N501Y), gamma (P.1 with mutations K417T, E484K, and N501Y), and delta (B.1.617.2 with mutations L452R, T478K, and P681R) variants have been emerging. These VOCs have raised concerns about potential increases in transmissibility, escape from vaccine-induced immunity, and decreased sensitivity to neutralization by convalescent sera or monoclonal antibodies, and increased disease severity [3–5].

SARS-CoV-2 has been estimated to have a mutation rate of approximately 8 × 10^−4^ nucleotides per site per year [6,7], which is a major mechanism of evolution in RNA viruses and increases the fitness in the host and transmissibility [8,9]. In this regard, understanding mutation dynamics that include variants with a low allele frequency that represent a subpopulation of viruses and consensus variants of the dominant viral population is required. Recent studies have shown intrahost genomic evolution and transmission patterns of SARS-CoV-2 during the course of infection [1,6,10–12]. These studies were conducted in patients within a month of infection or with prolonged infection but receiving COVID-19 treatment such as monoclonal antibody and convalescent plasma, which may encourage viral evolution [11]. Moreover, the samples studied were mainly from the respiratory tract.

In this study, we performed whole-genome sequencing of serial samples collected from various anatomic sites of two immunosuppressed patients with long-term SARS-CoV-2 shedding for 73 and 250 days, respectively, and without any specific treatment such as antiviral agent, monoclonal antibody or convalescent plasma treatment. We characterized the dynamics and diversity of SARS-CoV-2 over the prolonged course of infection and characterized household transmission from patient 2 to his mother, which occurred at least 38 days after the diagnosis of the infection in the patient.

## Materials and methods

### Patients and samples

Two patients with acute leukaemia (acute lymphoblastic leukaemia treated with B-cell targeting agents and chemotherapy, n = 1; acute myelogenous leukaemia treated with allogeneic haematopoietic stem cell transplant, n = 1) were confirmed with SARS-CoV-2 infection in September and November 2020 respectively, and hospitalised at tertiary hospitals in South Korea (Asan Medical Center and Seoul National University Hospital). One patient (Patient 1) presented with fever and had computed tomography (CT) feature of COVID-19 bronchopneumonia, while the other patient (Patient 2) had no respiratory symptoms other than nasal congestion. A total of 27 clinical samples were serially collected from two patients with laboratory-confirmed SARS-CoV-2 infection (nasopharyngeal swabs, n = 13; sputum, n = 6; throat swab, n = 1; saliva, n = 3; stool, n = 2; and urine, n = 1) and patient 2’s mother (nasopharyngeal swab, n = 1) at diagnosis (Table 1). All 27 samples were positive for SARS-CoV-2 by a real-time reverse-transcription polymerase chain reaction (rRT-PCR) targeting RNA-dependent RNA polymerase (RdRp) and envelope (E) genes. This study was approved by the institutional review board of Seoul National University Hospital (IRB No. H-2004-059-1116) and Asan Medical Center (IRB No. 2020–2560).

**Table 1.** Clinical samples and SARS-CoV-2 rRT-PCR assay in this study.

Patient	ID	Collection date	Day	Sample type	rRT-PCR (Ct, E/RdRP)
Patient 1	1-S1	03-Sep-2020	1	Sputum	12.92/14.97
	1-S2	03-Sep-2020	1	Nasopharyngeal swab	14.88/17.03
	1-S3	17-Sep-2020	15	Sputum	16.6/18.32
	1-S5	24-Sep-2020	22	Sputum	17.03/18.32
	1-S6	24-Sep-2020	22	Nasopharyngeal swab	17.88/19.37
	1-S7	12-Oct-2020	40	Nasopharyngeal swab	16.14/17.3
	1-S8	25-Oct-2020	53	Sputum	22.07/23.46
	1-S9	25-Oct-2020	53	Nasopharyngeal swab	16.41/17.06
	1-S12	12-Nov-2020	71	Sputum	20.12/21.26
	1-S17	05-Feb-2021	156	Sputum	26.21/26.04
Patient 2	2-S1	20-Nov-2020	0	Nasopharyngeal swab	12.22/11.55
	2-S2	02-Dec-2020	12	Nasopharyngeal swab	16.51/15.86
	2-R1	02-Dec-2020	12	Saliva	27.93/28.27
	2-S4	02-Dec-2020	12	Stool	24.83/25.17
	2-R4	07-Dec-2020	17	throat swab	18.93/18.23
	2-S6	09-Dec-2020	19	Nasopharyngeal swab	16.67/16.92
	2-S7	09-Dec-2020	19	Saliva	21.15/21.15
	2-S8	09-Dec-2020	19	Stool	20.64/21.83
	2-R2	09-Dec-2020	19	Urine	30.19/30.62
	2-S10	19-Dec-2020	29	Nasopharyngeal swab	20.05/19.24
	2-S11	28-Dec-2020	38	Nasopharyngeal swab	14.76/14.47
	2-S12	11-Jan-2021	52	Nasopharyngeal swab	21.55/20.44
	2-R8	13-Jan-2021	54	Saliva	24.05/24.09
	2-R10	18-Jan-2021	59	Nasopharyngeal swab	25.91/25.23
	2-R11	22-Jan-2021	63	Nasopharyngeal swab	23.96/23.75
	2-R12	26-Jan-2021	67	Nasopharyngeal swab	25.23/24.65
Patient 2’ mother	2-R3	11-Jan-2021		Nasopharyngeal swab	20.56/26.74

Day 0 was the day of clinical diagnosis. Cycle threshold (Ct) values <40 were defined as positive. Ct, Cycle threshold; rRT-PCR, real-time reverse-transcription PCR.

### Whole-genome sequencing

As previously described, whole-genome sequencing was performed using RNA extracted from clinical samples positive for SARS-CoV-2 by rRT-PCR [13]. RT-PCR was performed to obtain complementary DNA using the SuperScript III Reverse Transcriptase kit (Thermo Fisher Scientific, Waltham, MA, USA). Multiple overlapping PCR encompassing the full SARS-CoV-2 genome were performed, with an average fragment size of 800 base pairs (bp) (Table S1). Individual amplicons were pooled, of which 500 ng were used to prepare a library using the Nextera DNA Flex Library Prep kit (Illumina, San Diego, CA), according to the manufacturer’s instructions. Samples collected from patient 1 were processed and sequenced separately from other samples. Samples collected from patient 2 were sequenced in parallel with other samples, but each sample was individually barcoded. SARS-CoV-2 negative controls were not included in this study. Sequencing was performed using NextSeq 550 Sequencing System (Illumina), and sequencing data were then further processed to create consensus whole-genome sequences and to identify the variations. Alignment to the reference SARS-CoV-2 Wuhan-Hu-1 genome (GenBank NC_045512.2) was performed using the Burrows-Wheeler Aligner (BWA v.0.7.17) [14]. The variants were called and annotated using Samtools v.1.10 (Genome Research Limited, Cambridgeshire, United Kingdom) and SnpEff (v.5.0c), respectively [15]. To identify the variants, the criteria were applied as follows: (1) Sequencing depth ≥50, (2) base quality score ≥30, (3) minor allele read count ≥5 on each strand, and (4) variant allele frequency ≥5%. The Integrative Genomics Viewer was used to manually verify all variations (Broad Institute, Cambridge, MA, USA). Data obtained in this study were submitted to the Sequence Read Archive (SRA) (https://www.ncbi.nlm.nih.gov/sra) under accession PRJNA801401 [16].

### Phylogenetic analysis

A total of 8,096 SARS-CoV-2 genome sequences with complete sequence (>29,000 bases in length) and with high coverage (<1% Ns, undefined bases) collected from July 2020 to September 2021 with South Korea identifiers were downloaded from the GISAID database [17]. Due to large number of sequences available, we randomly subsampled this total dataset using Seqtk (https://github.com/lh3/seqtk) and obtained 149 sequences that matched the proportion of sequences and date range of sample collection as the total dataset (Table S2, Figure S1). Sequences were aligned using MAFFT v.7.475 (Osaka University, Osaka, Japan), and phylogenetic trees were inferred using the maximum likelihood method with the GTR+I+G4+F substitution model and 1000 bootstrap replicates using IQ-TREE v 1.6.1 open-source software (http://www.cibiv.at/software/iqtree). All trees were visualised using Figtree v.1.4.4 (Institute of Evolutionary Biology, University of Edinburgh, Edinburgh, United Kingdom) and TreeTime (https://github.com/neherlab/treetime).

### Genetic distance

We classified sample pairs into two subgroups: (1) intrahost sample pairs obtained at different time points and (2) intrahost sample pairs obtained from different anatomic sites. We then calculated the L1-norm genetic distance between each pair of samples. The frequencies of all four possible nucleotides (A, C, G, and T) between the two samples (p and q) on each genomic locus (k) were estimated as follows, where N represents the sum of all the distances across variable loci:

$$D=\sum_{k=1}^{N}\sum_{i=1}^{n}\left|p_{i}-q_{i}\right|$$

The genetic distances within each category of sample pairs were compared using the Mann – Whitney *U* test, which was calculated using SPSS version 19.0 (IBM Corp., Armonk, NY, USA). Statistical significance was set at *p* < 0.05.

### Transmission bottleneck estimation

For the household transmission pair, we estimated the transmission bottleneck size using a beta-binominal model (https://github.com/weissmanlab/BB_bottleneck) [10,18]. The maximum likelihood estimate with a 95% confidence interval was calculated.

### STR analysis

To confirm that the clinical specimens at the two different time points (at initial diagnosis and at day 156) were obtained from the identical patient (patient 1), STR analysis was performed using the AmpFLSTR® Identifiler® Plus kit, according to the manufacturer’s instructions. This assay used 16 STR markers, each with 2 to 28 alleles of different sizes. PCR products were analysed on an ABI 3730 Genetic Analyser (Applied Biosystems, Foster City, CA). Alleles for each STR marker were analysed with an ABI GeneScan analysis program (Applied Biosystems).

## Results

### Clinical presentation

Patient 1, a 20-year-old man with recurrent acute lymphoblastic leukaemia, was hospitalised and treated with blinatumomab, inotuzumab ozogamicin, and chemotherapy (FLAG-IDA). During his hospitalisation, a nosocomial outbreak of COVID-19 occurred. He had close contact with a COVID-19 patient on 28 August 2020 and was confirmed with SARS-CoV-2 infection on 2 September 2020 (Table S3). He developed a fever lasting 7 days (4 September 2020–10 September 2020), and chest CT in patient 1 was suggestive of bronchopneumonia. He received antibiotics and symptomatic care as needed. He had prolonged SARS-CoV-2 shedding through day 250 and continued pneumonia. Due to clinical deterioration, he died on day 263.

Patient 2, a 25-year-old man who had a history of recurrent acute myelogenous leukaemia who had undergone allogeneic haematopoietic stem cell transplant 1 year previously, developed chronic graft‐versus‐host disease and Evans syndrome, thus treated with immunosuppressive agents (mycophenolate mofetil (MMF) and prednisone), rituximab, and romiplostim. He had close contact with a COVID-19 patient on 13 November 2020, tested positive for SARS-CoV-2 on 20 November 2020, and was admitted to hospital (Table S3). He reported no respiratory symptoms other than nasal congestion without any abnormal findings on chest radiography. During his hospitalisation, he had an intermittent fever every second day. Lung lesions were consistently absent on chest radiography, and he had no respiratory symptoms; thus, his fever was considered unlikely to be a COVID-19 related symptom. He was provided with symptomatic care, as needed, and received prednisone and MMF as part of the treatment regimen for his underlying disease. He was discharged on day 26 (16 December 2020), according to the symptom-based strategy for discontinuing isolation of persons with COVID-19. After discharge, he was quarantined at home but visited the hospital every 4–5 days for SARS-CoV-2 monitoring by rRT-PCR and for receiving treatment for his underlying disease. He had prolonged SARS-CoV-2 shedding through day 73. His mother, who was his primary caregiver, reported having respiratory symptoms and tested positive for SARS-CoV-2 by rRT-PCR on day 52 of patient 2’s illness.

### SARS-CoV-2 consensus genome sequences

The genome sequences from each sample covered a median 99.56 % (Quartile 1-Quartile 3, 99.48% −99.62%) of the 29,903 bp reference sequences, with an average depth of 10,015×. Summary statistics of the whole-genome sequencing data are presented in Table S4. SARS-CoV-2 consensus genome sequences were obtained from all 27 samples: All sequences except the sequence from patient 1 on day 156 (B.1.1.122 pangolin lineage) were identified as B.1.497 pangolin lineage by pangolin COVID-19 Lineage Assigner (version 3.1.17) (https://pangolin.cog-uk.io/).

**Figure 1.**
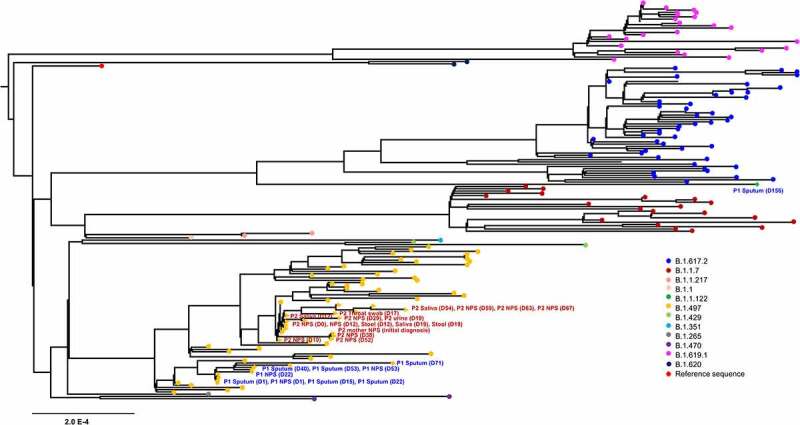
Phylogenetic analysis of severe acute respiratory syndrome coronavirus 2 strains. A maximum likelihood phylogenetic tree was generated with 27 sequences from this study (yellow, *n* = 26; green, *n* = 1) and 149 SARS-CoV-2 genomes obtained from GISAID. All of the SARS-CoV-2 genomes in South Korea were downloaded from the GISAID database, and 149 sequences that matched the proportion of sequences and date range of sample collection as the total dataset were obtained. Diamond and triangle at each branch tip represent samples from patient 1 and patient 2, respectively.

We then performed maximum-likelihood phylogenetic analysis of patient sequences (n = 27) obtained in this study, along with publically available 149 sequences from GISAID. SARS-CoV-2 genome sequences at diagnosis from our patients clustered with sequences of B.1.497 pangolin lineages that were most prevalent in Korea (Figure 1, Figure S2). Remarkably, the sequence on day 156 (5 February 2021) from patient 1 had 101 newly emerged variants (VAF, 14.93–100%), of which 14 were present within spike protein (L10L, T19R, G404S, N501Y, N536D, N556D, A570D, I584V, P681R, T734T, D950N, K1045E, K1086K, and C1243C) (Table S5). This sequence fell into B.1.1.122 pango lineage and we observed that this lineage was closely related to VOC delta (B.1.617.2) variant collected in Korea between June 2021 and September 2021. Considering that the first case of P681R-containing SARS-CoV-2 consensus genome sequence was detected in Korea at the end of April 2021 (Figure S3), this finding suggests that nucleotide changes that have not been detected yet in the population may be arisen intrahost during the course of infection.

### Temporal dynamics of intrahost SARS-CoV-2 variants

**Figure 2.**
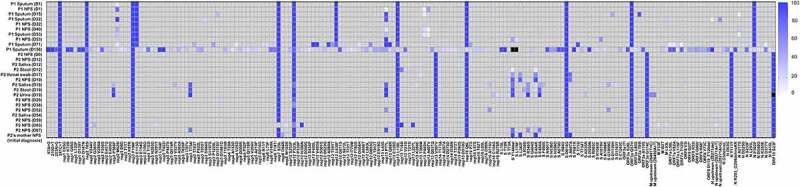
Heatmap representing SARS-CoV-2 variant allele frequency of each variant detected in the samples. Variants that were not detected because of low coverage are shown in black.

**Figure 3.**
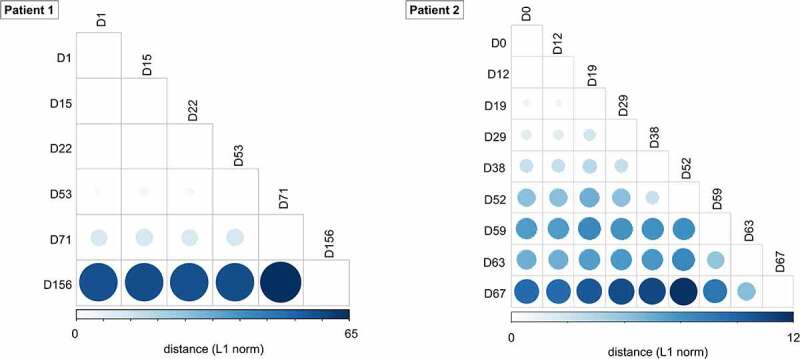
Pairwise genetic distance among serial SARS-CoV-2 respiratory samples during the course of infection. The L1 norm genetic distances are shown by a colour gradient and size difference. Sputum specimens (patient 1) and nasopharyngeal swabs (patient 2) for each time point were analysed.

We further examined intrahost SARS-CoV-2 variants in longitudinal respiratory samples from patients. In respiratory samples at diagnosis, consensus sequences from two patients shared 10 out of 16 consensus variants 5’UTR 241C>T, nsp2 T85I, nsp3 F106F, nsp7 S25L, nsp12 P323L, nsp13 L138L, nsp16 Q6L, S D614G, ORF3a Q57H, N P302P), which defined two consensus sequences as same lineage (i.e. B.1.497) (Figure 2, Table S5). However, intrahost variants that emerged during the course of infection showed diversity between individual hosts. We observed 178 intrahost variants, of which only three variants (nsp13 P77L, S Y144del, and M L93L) were shared in two patients. The pairwise genetic distance among longitudinal respiratory samples increased over time since the diagnosis (Figure 3).

**Figure 4.**
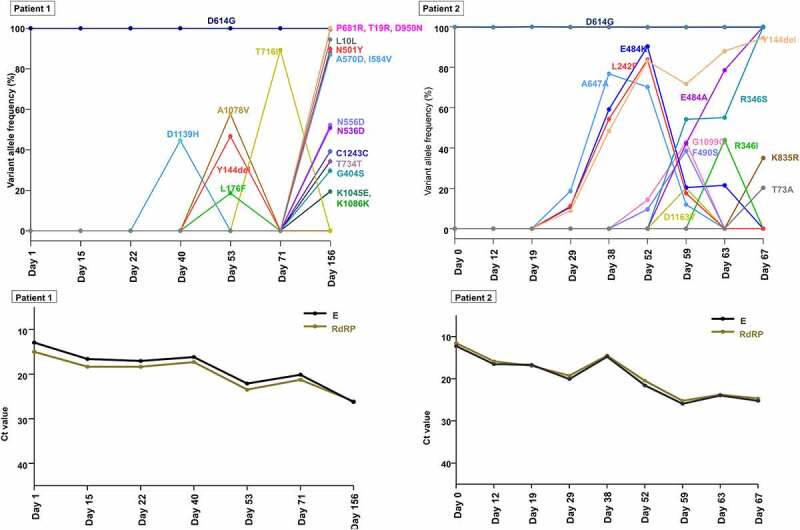
Temporal dynamics of SARS-CoV-2 variants within spike gene and corresponding viral loads during the patient’s course of infection in each patient (A and C, patient 1; B and D, patient 2). A lower cycle threshold (Ct) value corresponds to a higher viral load.

Notably, we observed several interesting mutations in the spike protein of SARS-CoV-2 sequences from each host. In patient 1, N501Y and P681R, signature mutations characterizing the alpha (B.1.1.7) and delta (B.1.617.2) variants, respectively, appeared on day 156 with frequencies of 89.9% and 100.0%, respectively (Figure 4). Additional spike protein mutations frequently accumulated in either alpha (B.1.1.7) or delta (B.1.617.2) variants were also observed: A570D (alpha [lineage B.1.1.7], frequency of 88.2%), T19R (delta [lineage B. 1.617.2], frequency of 100.0%), and D950N (delta [lineage B. 1.617.2], frequency of 99.57%) on day 156. To exclude the possibility of contamination or specimen switch, we performed short tandem repeat (STR) analysis (Figure S4). The results of all 16 STR markers showed identical allele size between two specimens (at initial diagnosis and at day 156), which excluded contamination by nucleic acid from different host and also confirmed that these clinical specimens at the two different time points were collected from the identical patient (i.e. patient 1).

In patient 2, E484K and Y144del appeared at low frequencies (10.70% and 8.90%, respectively) on day 29, increased and reached a frequency of 83.1% and 90.36%, respectively, on day 52. Subsequently, E484K decreased to a frequency of 20.42% (day 59), while Y144del increased to a frequency of 94.5% (day 67). E484A, another amino acid change at residue 484, increased from undetectable on day 52% to 42.2% on day 59. The sequencing reads that contained E484A were completely mutually exclusive with the reads containing E484K (Figure S5). The frequency of E484A continued to increase after day 59, while the frequency of E484K continued to decrease and was completely replaced by E484A from day 67. R346S and R346I were present on day 63 at frequencies of 54.21% and 48.83%, respectively. These two variants were mutually exclusive substitutions at the residue of 346 (Figure S6), and R346I was outcompeted by R346S. These trends in allele frequencies of spike gene variants suggest continuous competition between viral populations with different variants.

### SARS-CoV-2 mutations within different tissue samples

**Figure 5.**
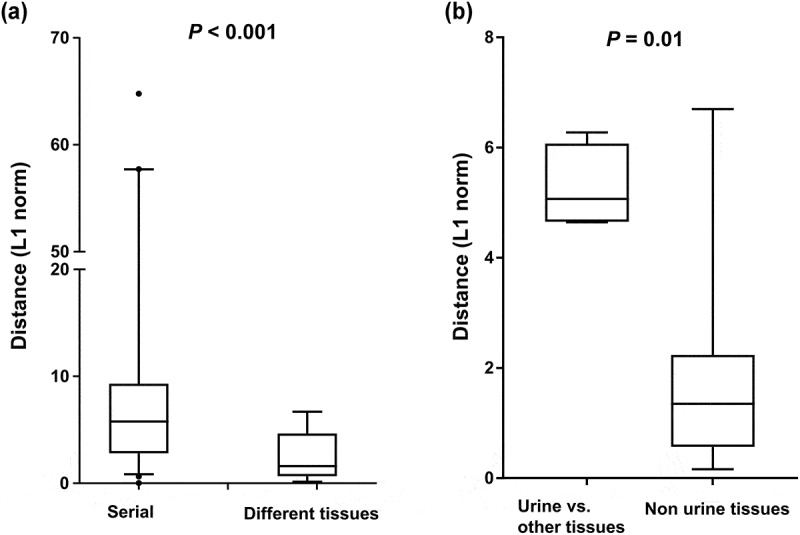
Genetic distance between sample pairs. (a) Sample pairs were classified into three subgroups: (1) intrahost sample pairs obtained at different time points, (2) intrahost sample pairs obtained from different anatomic sites. (b) the sample pairs were categorized into urine/non-urine sample pairs and non-urine/non-urine sample pairs. Box (median ± 25%) and whisker (5% and 95%) plots represent the distribution of the genetic distances (L1 norm) between sample pairs.

During the infection, SARS-CoV-2 RNA was also detected in samples from anatomic sites other than the respiratory tract, including stool, urine, and saliva. SARS-CoV-2 genomic diversity among different tissue samples appeared to be randomly distributed at various loci across the genome (Figure 2). The genetic distance between the different tissue pairs was significantly lower than that of temporally different sample pairs (*P* < 0.001, Figure 5(a)). We further estimated the genetic distance between sample pairs from different anatomic sites on the same day. The sample pairs were categorized into urine/non-urine sample pairs and non-urine/non-urine sample pairs. The genetic distances between urine and non-urine samples were significantly greater than those between non-urine and non-urine samples (*P* = 0.01; Figure 5(b)). The urine sample carried 7 unique substitutions (nsp2; G265V, nsp3; N291Y, nsp8; W182R, nsp12; N386N, nsp13; L227L, M; L93L, upstream of N; A28271T) that were not observed in non-urine samples across the genome (Figure 2, Table S5).

### Long-term infectious viral shedding and intra-household transmission

Patient 2’s mother, who was his primary caregiver, reported having respiratory symptoms on day 48 and tested positive for SARS-CoV-2 on rRT-PCR on day 52. We compared the first positive sample from the patient’s mother with a sample collected from the patient on day 38 (Figure 2, Table S5). We confirmed that the consensus sequences of viral RNA from his mother did not differ from the virus genomic profile of the patient’s sample, which enabled us to infer that direct transmission from patient 2 to his mother occurred after day 38. The transmission bottleneck size was estimated to be 3 (Table S6).

## Discussion

In this study, we investigated the shedding dynamics and diversity of SARS-CoV-2 during the course of infection in immunosuppressed patients who had long-term viral shedding in the absence of specific COVID-19 treatment. We further described a household transmission from patient 2 to his mother, which occurred at least 38 days after the diagnosis.

The spectrum of intrahost variants and the time at which each mutation appeared throughout prolonged infection differed for each host. In each host, viruses may differently mutate and adjust to the selective pressure of the host environment, which could enable the virus to replicate efficiently for fitness in each host.

The frequency of variants continuously changed, and there was a turnover of the dominant viral population. These findings are comparable to those of recent reports [12,19], except that the patient in this study did not receive COVID-19-specific treatment. Previous reports suggested that the use of monoclonal antibodies or convalescent plasma can contribute to viral evolution [12,19,20]. In this study, we further identified a broad spectrum of SARS-CoV-2 evolution even without SARS-CoV-2-targeted treatment

N501Y, P681R, and E484K emerged *in vivo* during infection and became the dominant population. N501Y, shared by major VOCs alpha (B.1.1.7), beta (B.1.351), and gamma (P.1), occurs in the receptor-binding domain region of spike gene. N501Y exhibits increased binding affinity to human angiotensin-converting enzyme-2 (ACE2) receptors, which may increase transmissibility [21]. P681R, contained by VOC delta (B.1.617.2) variant, occurs in the furin cleavage site. P681R increases the fusion of membrane in the respiratory tract, which may be attributed to the increase in transmissibility and pathogenicity in the infected host [22,23]. E484K is the variant present in the beta (B.1.351) and gamma (P.1) variants and exhibits increased binding affinity to human ACE2 receptors [24,25]. It has been reported that the single E484K variant affects the binding of neutralizing antibodies and decreases the neutralising activity of convalescent and post-vaccination sera [26,27]. In our patient, the generation of neutralising antibodies may not have been sufficient due to the compromised immune status, or the E484K variant may render the SARS-CoV-2 having reduced susceptibility to neutralizing antibodies in the early stages of infection, which probably contributed to prolonged viral shedding in this patient.

It is notable that P681R, not detected in the publicly available genome in Korea before the end of April 2021, was observed from patient 1 on 5 February 2021, which suggests that this nucleotide changes may be arisen intrahost during the course of infection. The variants that arose *de novo* within hosts and reached dominant viral strains during the infection period could be candidate variants that are likely to spread to the population eventually. Therefore, it might be necessary to take pre-emptive measures after examining the effect of these variants on the effects of vaccines and therapeutic antibodies.

Viral RNA from day 156 of patient 1 was clustered into distinct lineage from other samples of patient 1. Also, N501Y has been already present at low levels in publicly available genome in Korea, and P681R, not detected in any genomes in Korea in February 2021, has been detected globally as early as November 2020. Moreover, it would be possible that genomes containing N501Y and P681R might have existed but not have been sampled and sequenced during genome surveillance in Korea [28]. Regarding the rate of evolution within host over time in patient 1, many consensus mutations emerged and disappeared, and faster rate of evolution was observed in the second half of infection compared with first half of infection in patient 1. Thus, the possibility that patient 1 was infected with a new SARS-CoV-2 strain cannot be totally excluded.

Recent reports suggest that the kidneys can be directly infected with SARSCoV2, and proximal tubule epithelial cells, where ACE2 is strongly expressed, are most likely associated with the infection [29–33]. However, the genomic characteristics of SARSCoV2 in urine have not been fully described. In this study, unique genetic changes were observed across the genome in urine samples that were not observed in other sample types, and a high genetic distance was observed between the urine and non-urine sample pairs. We speculate that substitutions were acquired during active replication of SARS-CoV-2 in proximal tubule cells, possibly due to less stringent selection pressure in the kidney environment [6]. The genetic diversity according to the anatomic site needs further research in order to understand the tissue-specific pressure for viral adaptation.

This study has several limitations. First, this study included a small number of patients, limiting the generalizability of the study results. However, this study analysed serial samples collected from various anatomic sites of patients, which demonstrated the temporal dynamics and genetic diversity of SARS-CoV-2 during the entire course of infection. Second, negative control to detect contamination was not sequenced in parallel. However, STR analysis was performed, which excluded contamination by nucleic acid from different host. Our findings may provide new insights into the dynamics of SARS-CoV-2 in response to interactions between the virus and host.

## Supplementary Material

Supplemental Material

## Data Availability

Data obtained in this study were submitted to the Sequence Read Archive (SRA) (https://doi.org/10.1080/21505594.2022.2101198) under accession PRJNA801401.
