# Supplementary material for: SARS-CoV-2 shedding dynamics and transmission in immunosuppressed patients
Source: Virulence. 2022 Jul 26;13(1):1242–51. doi: 10.1080/21505594.2022.2101198 (PMC9336477; doi:10.1080/21505594.2022.2101198)
Supplement: Supplemental Material [file KVIR_A_2101198_SM8377.zip › supplementary/4. R2_supple table 5_covid19_dynamics_Virulence_220128.docx]

| **position** | **REF** | **ALT** | **Gene** | **Protein** | **Amino acid change** | **Patient 1** | | | | | | | | | | **Patient 2** | | | | | | | | | | | | | | | | **Patient 2 mother** |
| --- | --- | --- | --- | --- | --- | --- | --- | --- | --- | --- | --- | --- | --- | --- | --- | --- | --- | --- | --- | --- | --- | --- | --- | --- | --- | --- | --- | --- | --- | --- | --- | --- |
|  |  |  |  |  |  | **D1** | | **D15** | **D22** | | **D40** | **D53** | | **D71** | **D156** | **D0** | **D12** | | | **D19** | | | | | **D29** | **D38** | **D52** | | **D59** | **D63** | **D67** | **Initial diagnosis** |
|  |  |  |  |  |  | **sputum** | **NP swab** | **sputum** | **sputum** | **NP swab** | **NP swab** | **sputum** | **NP swab** | **sputum** | **sputum** | **NP swab** | **NP swab** | **saliva** | **stool** | **throat swab** | **NP swab** | **saliva** | **stool** | **urine** | **NP swab** | **NP swab** | **NP swab** | **saliva** | **NP swab** | **NP swab** | **NP swab** | **NP swab** |
| 97 | A | G |  | 5'UTR |  |  |  |  |  |  |  |  |  |  | 100.00 |  |  |  |  |  |  |  |  |  |  |  |  |  |  |  |  |  |
| 210 | G | T |  | 5'UTR |  |  |  |  |  |  |  |  |  |  | 100.00 |  |  |  |  |  |  |  |  |  |  |  |  |  |  |  |  |  |
| 223 | A | G |  | 5'UTR |  |  |  |  |  |  |  |  |  |  | 81.82 |  |  |  |  |  |  |  |  |  |  |  |  |  |  |  |  |  |
| 241 | C | T |  | 5'UTR |  | 99.59 | 100.00 | 99.60 | 100.00 | 99.60 | 100.00 | 100.00 | 100.00 | 94.44 | 100.00 | 99.79 | 99.73 | 99.62 | 99.73 | 99.44 | 99.86 | 99.78 | 99.66 | 99.36 | 99.83 | 99.76 | 99.85 | 99.46 | 99.68 | 99.53 | 99.28 | 99.48 |
| 459 | A | G | ORF1a | nsp1 | E65G |  |  |  |  |  |  |  |  | 22.92 |  |  |  |  |  |  |  |  |  |  |  |  |  |  |  |  |  |  |
| 480 | A | G | ORF1a | nsp1 | K72R |  |  |  |  |  |  |  |  |  | 97.78 |  |  |  |  |  |  |  |  |  |  |  |  |  |  |  |  |  |
| 557 | G | A | ORF1a | nsp1 | G98S |  |  |  |  |  |  |  |  |  | 91.89 |  |  |  |  |  |  |  |  |  |  |  |  |  |  |  |  |  |
| 629 | C | T | ORF1a | nsp1 | L122F |  |  |  |  |  |  | 25.94 |  |  |  |  |  |  |  |  |  |  |  |  |  |  |  |  |  |  |  |  |
| 680 | G | T | ORF1a | nsp1 | D139Y |  |  |  |  |  |  |  |  |  | 93.75 |  |  |  |  |  |  |  |  |  |  |  |  |  |  |  |  |  |
| 704 | G | A | ORF1a | nsp1 | D147N |  |  |  |  |  |  |  |  |  | 96.15 |  |  |  |  |  |  |  |  |  |  |  |  |  |  |  |  |  |
| 1059 | C | T | ORF1a | nsp2 | T85I | 99.59 | 100.00 | 99.59 | 100.00 | 99.15 | 99.60 | 99.18 | 100.00 | 100.00 | 68.02 | 99.83 | 99.65 | 99.56 | 99.70 | 99.59 | 99.69 | 99.61 | 99.76 | 99.66 | 99.53 | 99.66 | 99.73 | 99.69 | 99.77 | 99.69 | 99.42 | 99.55 |
| 1537 | T | G | ORF1a | nsp2 | V244V |  |  |  |  |  |  |  |  |  | 33.33 |  |  |  |  |  |  |  |  |  |  |  |  |  |  |  |  |  |
| 1599 | G | T | ORF1a | nsp2 | G265V |  |  |  |  |  |  |  |  |  |  |  |  |  |  |  |  |  |  | 36.42 |  |  |  |  |  |  |  |  |
| 1656 | T | C | ORF1a | nsp2 | V284A |  |  |  |  |  |  |  |  |  | 16.91 |  |  |  |  |  |  |  |  |  |  |  |  |  |  |  |  |  |
| 1872 | T | C | ORF1a | nsp2 | F356S |  |  |  |  |  |  |  |  |  | 76.11 |  |  |  |  |  |  |  |  |  |  |  |  |  |  |  |  |  |
| 2175 | A | G | ORF1a | nsp2 | E457G |  |  |  |  |  |  |  |  |  | 26.48 |  |  |  |  |  |  |  |  |  |  |  |  |  |  |  |  |  |
| 2184 | A | G | ORF1a | nsp2 | E460G |  |  |  |  |  |  |  |  |  | 29.09 |  |  |  |  |  |  |  |  |  |  |  |  |  |  |  |  |  |
| 2509 | C | T | ORF1a | nsp2 | P568P |  |  |  |  |  |  |  |  |  |  |  |  |  |  |  |  |  |  |  |  |  |  |  | 36.42 | 91.01 | 99.65 |  |
| 2683 | G | T | ORF1a | nsp2 | M626I |  | 10.61 | 43.65 | 31.17 | 77.96 |  |  | 100.00 | 12.07 |  |  |  |  |  |  |  |  |  |  |  |  |  |  |  |  |  |  |
| 2746 | T | C | ORF1a | nsp3 | D9D |  |  |  |  |  |  |  |  |  | 15.50 |  |  |  |  |  |  |  |  |  |  |  |  |  |  |  |  |  |
| 2752 | T | C | ORF1a | nsp3 | T11T |  |  |  |  |  |  |  |  |  | 21.58 |  |  |  |  |  |  |  |  |  |  |  |  |  |  |  |  |  |
| 3009 | A | G | ORF1a | nsp3 | K97R |  |  |  |  |  |  |  |  |  | 23.73 |  |  |  |  |  |  |  |  |  |  |  |  |  |  |  |  |  |
| 3037 | C | T | ORF1a | nsp3 | F106F | 98.64 | 100.00 | 97.87 | 97.92 | 98.76 | 98.60 | 100.00 | 98.29 | 99.57 | 99.10 | 99.50 | 99.41 | 99.54 | 99.11 | 99.59 | 99.63 | 99.77 | 99.48 | 99.53 | 99.53 | 99.38 | 99.64 | 99.63 | 97.00 | 99.79 | 97.88 | 99.89 |
| 3057 | A | G | ORF1a | nsp3 | E113G | 99.59 | 100.00 | 100.00 | 100.00 | 100.00 | 99.60 | 100.00 | 100.00 | 91.70 |  |  |  |  |  |  |  |  |  |  |  |  |  |  |  |  |  |  |
| 3180 | A | G | ORF1a | nsp3 | E154G |  |  |  |  |  |  |  |  |  | 25.52 |  |  |  |  |  |  |  |  |  |  |  |  |  |  |  |  |  |
| 3186 | A | G | ORF1a | nsp3 | E156G |  |  |  |  |  |  |  |  |  | 18.81 |  |  |  |  |  |  |  |  |  |  |  |  |  |  |  |  |  |
| 3267 | C | T | ORF1a | nsp3 | T183I |  |  |  |  |  |  |  |  |  | 71.67 |  |  |  |  |  |  |  |  |  |  |  |  |  |  |  |  |  |
| 3506 | A | G | ORF1a | nsp3 | N263D |  |  |  |  |  |  |  |  |  | 21.70 |  |  |  |  |  |  |  |  |  |  |  |  |  |  |  |  |  |
| 3590 | A | T | ORF1a | nsp3 | N291Y |  |  |  |  |  |  |  |  |  |  |  |  |  |  |  |  |  |  | 21.00 |  |  |  |  |  |  |  |  |
| 3809 | C | T | ORF1a | nsp3 | L364F |  |  |  |  |  |  |  |  |  | 99.38 |  |  |  |  |  |  |  |  |  |  |  |  |  |  |  |  |  |
| 3984 | C | T | ORF1a | nsp3 | T422I |  |  |  |  |  |  |  |  |  | 99.48 |  |  |  |  |  |  |  |  |  |  |  |  |  |  |  |  |  |
| 4058 | C | T | ORF1a | nsp3 | P447S |  |  |  |  |  |  |  |  |  |  |  |  |  |  |  |  |  |  |  |  |  |  |  | 25.46 |  |  |  |
| 4260 | A | G | ORF1a | nsp3 | Q514R |  |  |  |  |  |  |  |  |  | 31.40 |  |  |  |  |  |  |  |  |  |  |  |  |  |  |  |  |  |
| 4300 | G | T | ORF1a | nsp3 | V527V |  |  |  |  |  |  |  |  |  |  |  |  |  |  |  |  |  |  |  |  |  |  |  | 38.31 |  |  |  |
| 4405 | A | T | ORF1a | nsp3 | A562A |  |  |  |  |  |  |  |  |  | 37.94 |  |  |  |  |  |  |  |  |  |  |  |  |  |  |  |  |  |
| 4595 | A | C | ORF1a | nsp3 | T626P |  |  |  |  |  |  |  |  | 22.92 |  |  |  |  |  |  |  |  |  |  |  |  |  |  |  |  |  |  |
| 4838 | A | G | ORF1a | nsp3 | I707V |  |  |  |  |  |  |  |  |  | 31.85 |  |  |  |  |  |  |  |  |  |  |  |  |  |  |  |  |  |
| 4980 | C | T | ORF1a | nsp3 | T754I |  |  |  |  |  |  |  |  |  |  |  |  |  |  |  |  |  |  |  |  |  |  | 72.99 | 83.28 | 85.93 | 99.41 |  |
| 5008 | G | T | ORF1a | nsp3 | T763T |  |  |  |  |  |  |  |  | 40.94 |  |  |  |  |  |  |  |  |  |  |  |  |  |  |  |  |  |  |
| 5184 | C | T | ORF1a | nsp3 | P822L |  |  |  |  |  |  |  |  |  | 41.15 |  |  |  |  |  |  |  |  |  |  |  |  |  |  |  |  |  |
| 5584 | A | G | ORF1a | nsp3 | T955T |  |  |  |  |  |  |  |  |  | 47.30 |  |  |  |  |  |  |  |  |  |  |  |  |  |  |  |  |  |
| 5671 | A | G | ORF1a | nsp3 | S984S |  |  |  |  |  |  |  | 21.97 |  |  |  |  |  |  |  |  |  |  |  |  |  |  |  |  |  |  |  |
| 6310 | C | T | ORF1a | nsp3 | S1197S |  |  |  |  |  |  |  |  |  |  |  |  |  |  |  |  |  |  |  |  |  | 24.79 |  |  |  |  |  |
| 6402 | C | T | ORF1a | nsp3 | P1228L |  |  |  |  |  |  |  |  | 50.00 |  |  |  |  |  |  |  |  |  |  |  |  |  |  |  |  |  |  |
| 6672 | A | T | ORF1a | nsp3 | D1318V |  |  |  |  |  | 36.56 |  |  |  |  |  |  |  |  |  |  |  |  |  |  |  |  |  |  |  |  |  |
| 7162 | C | T | ORF1a | nsp3 | D1481D |  |  |  |  |  |  |  |  |  |  |  |  | 24.33 |  |  |  |  |  |  |  |  |  |  |  |  |  |  |
| 8208 | C | T | ORF1a | nsp3 | T1830I |  |  |  |  |  |  |  |  |  | 38.64 |  |  |  |  |  |  |  |  |  |  |  |  |  |  |  |  |  |
| 8636 | A | G | ORF1a | nsp4 | T28A |  |  |  |  |  |  |  |  |  | 25.74 |  |  |  |  |  |  |  |  |  |  |  |  |  |  |  |  |  |
| 9012 | C | A | ORF1a | nsp4 | A153D |  |  |  |  |  |  |  |  |  | 16.15 |  |  |  |  |  |  |  |  |  |  |  |  |  |  |  |  |  |
| 9431 | G | A | ORF1a | nsp4 | V293I |  |  |  |  |  |  |  |  | 10.00 |  |  |  |  |  |  |  |  |  |  |  |  |  |  |  |  |  |  |
| 10053 | A | G | ORF1a | nsp4 | Q500R |  |  |  |  |  |  |  |  |  | 32.19 |  |  |  |  |  |  |  |  |  |  |  |  |  |  |  |  |  |
| 10210 | T | C | ORF1a | nsp5 | P52P |  |  |  |  |  |  |  |  |  | 21.43 |  |  |  |  |  |  |  |  |  |  |  |  |  |  |  |  |  |
| 10383 | A | G | ORF1a | nsp5 | Q110R |  |  |  |  |  |  |  |  |  | 17.09 |  |  |  |  |  |  |  |  |  |  |  |  |  |  |  |  |  |
| 10666 | T | G | ORF1a | nsp5 | V204V |  |  |  |  |  |  |  |  |  | 28.27 |  |  |  |  |  |  |  |  |  |  |  |  |  |  |  |  |  |
| 10682 | G | C | ORF1a | nsp5 | A210P |  |  |  |  |  |  |  |  |  | 23.12 |  |  |  |  |  |  |  |  |  |  |  |  |  |  |  |  |  |
| 10701 | A | G | ORF1a | nsp5 | D216G |  |  |  |  |  |  |  |  |  | 19.43 |  |  |  |  |  |  |  |  |  |  |  |  |  |  |  |  |  |
| 11003 | C | T | ORF1a | nsp6 | H11Y |  |  |  |  |  |  |  |  | 20.00 |  |  |  |  |  |  |  |  |  |  |  |  |  |  |  |  |  |  |
| 11195 | C | T | ORF1a | nsp6 | L75F |  |  |  |  |  |  |  |  | 18.87 |  |  |  |  |  |  |  |  |  |  |  |  |  |  |  |  |  |  |
| 11418 | T | C | ORF1a | nsp6 | V149A |  |  |  |  |  |  |  |  |  | 74.09 |  |  |  |  |  |  |  |  |  |  |  |  |  |  |  |  |  |
| 11514 | C | T | ORF1a | nsp6 | T181I |  |  |  |  |  |  |  |  |  | 67.63 |  |  |  |  |  |  |  |  |  |  |  |  |  |  |  |  |  |
| 11916 | C | T | ORF1a | nsp7 | S25L | 99.59 | 100.00 | 98.77 | 100.00 | 100.00 | 100.00 | 100.00 | 99.18 | 99.59 | 92.59 | 99.86 | 99.85 | 99.80 | 99.86 | 99.83 | 99.80 | 99.72 | 99.83 |  | 99.74 | 99.86 | 99.77 | 100.00 | 99.74 | 99.93 | 99.56 | 99.75 |
| 12281 | G | A | ORF1a | nsp8 | D64N |  |  |  |  |  |  |  |  |  |  |  |  |  |  |  |  |  |  |  |  |  |  |  |  |  |  | 49.03 |
| 12635 | T | A | ORF1a | nsp8 | W182R |  |  |  |  |  |  |  |  |  |  |  |  |  |  |  |  |  |  | 17.18 |  |  |  |  |  |  |  |  |
| 14203 | T | C | ORF1b | nsp12 | S255P |  |  |  |  |  |  |  |  |  | 37.07 |  |  |  |  |  |  |  |  |  |  |  |  |  |  |  |  |  |
| 14408 | C | T | ORF1b | nsp12 | P323L | 97.95 | 96.51 | 95.85 | 94.25 | 94.71 | 98.35 | 97.01 | 96.57 | 99.02 | 79.82 | 96.40 | 96.73 | 82.19 | 57.55 | 95.16 | 96.14 | 97.13 | 95.46 | 63.77 | 95.46 | 95.55 | 96.65 | 97.37 | 72.76 | 92.09 | 100.00 | 93.78 |
| 14457 | A | G | ORF1b | nsp12 | P339P |  |  |  |  |  |  |  |  |  | 30.73 |  |  |  |  |  |  |  |  |  |  |  |  |  |  |  |  |  |
| 14598 | T | C | ORF1b | nsp12 | N386N |  |  |  |  |  |  |  |  |  |  |  |  |  |  |  |  |  |  | 96.59 |  |  |  |  |  |  |  |  |
| 14609 | A | G | ORF1b | nsp12 | D390G |  |  |  |  |  |  |  |  |  | 23.75 |  |  |  |  |  |  |  |  |  |  |  |  |  |  |  |  |  |
| 14740 | T | C | ORF1b | nsp12 | S434P |  |  |  |  |  |  |  |  |  | 17.91 |  |  |  |  |  |  |  |  |  |  |  |  |  |  |  |  |  |
| 14878 | T | C | ORF1b | nsp12 | F480L |  |  |  |  |  |  |  |  | 100.00 |  |  |  |  |  |  |  |  |  |  |  |  |  |  |  |  |  |  |
| 14918 | T | C | ORF1b | nsp12 | V493A |  |  |  |  |  |  |  |  | 100.00 |  |  |  |  |  |  |  |  |  |  |  |  |  |  |  |  |  |  |
| 14943 | A | G | ORF1b | nsp12 | S501S |  |  |  |  |  |  |  |  |  | 27.08 |  |  |  |  |  |  |  |  |  |  |  |  |  |  |  |  |  |
| 15008 | A | G | ORF1b | nsp12 | D523G |  |  |  |  |  |  |  |  |  | 31.51 |  |  |  |  |  |  |  |  |  |  |  |  |  |  |  |  |  |
| 15180 | C | T | ORF1b | nsp12 | A580A |  |  |  |  |  |  |  |  | 93.33 |  |  |  |  |  |  |  |  |  |  |  |  |  |  |  |  |  |  |
| 15213 | A | G | ORF1b | nsp12 | T591T |  |  |  |  |  |  |  |  | 55.71 |  |  |  |  |  |  |  |  |  |  |  |  |  |  |  |  |  |  |
| 15352 | C | T | ORF1b | nsp12 | L638F | 99.16 | 98.67 | 99.58 | 99.54 | 99.58 | 99.14 | 100.00 | 99.09 | 80.00 |  |  |  |  |  |  |  |  |  |  |  |  |  |  |  |  |  |  |
| 15451 | G | A | ORF1b | nsp12 | G671S |  |  |  |  |  |  |  |  |  | 79.19 |  |  |  |  |  |  |  |  |  |  |  |  |  |  |  |  |  |
| 15455 | C | T | ORF1b | nsp12 | S672L |  |  |  |  |  |  |  |  |  | 22.77 |  |  |  |  |  |  |  |  |  |  |  |  |  |  |  |  |  |
| 15543 | G | T | ORF1b | nsp12 | T701T |  |  |  |  |  |  |  |  |  | 19.64 |  |  |  |  |  |  |  |  |  |  |  |  |  |  |  |  |  |
| 15777 | A | G | ORF1b | nsp12 | I779M |  |  |  |  |  |  |  |  |  | 14.93 |  |  |  |  |  |  |  |  |  |  |  |  |  |  |  |  |  |
| 15785 | T | C | ORF1b | nsp12 | F782S |  |  |  |  |  |  |  |  |  | 20.18 |  |  |  |  |  |  |  |  |  |  |  |  |  |  |  |  |  |
| 15850 | G | C | ORF1b | nsp12 | D804H |  |  |  |  |  |  |  |  | 15.56 |  |  |  |  |  |  |  |  |  |  |  |  |  |  |  |  |  |  |
| 16036 | G | A | ORF1b | nsp12 | A866T |  |  |  |  |  |  |  |  |  | 31.86 |  |  |  |  |  |  |  |  |  |  |  |  |  |  |  |  |  |
| 16089 | G | A | ORF1b | nsp12 | L883L |  |  |  |  |  |  |  |  |  | 41.26 |  |  |  |  |  |  |  |  |  |  |  |  |  |  |  |  |  |
| 16118 | A | G | ORF1b | nsp12 | D893G |  |  |  |  |  |  |  |  | 59.38 |  |  |  |  |  |  |  |  |  |  |  |  |  |  |  |  |  |  |
| 16176 | T | C | ORF1b | nsp12 | T912T |  |  |  |  |  |  |  |  |  | 44.76 |  |  |  |  |  |  |  |  |  |  |  |  |  |  |  |  |  |
| 16196 | A | G | ORF1b | nsp12 | E919G |  |  |  |  |  |  |  |  |  | 45.02 |  |  |  |  |  |  |  |  |  |  |  |  |  |  |  |  |  |
| 16364 | T | C | ORF1b | nsp13 | L43S |  |  |  |  |  |  |  |  |  | 48.86 |  |  |  |  |  |  |  |  |  |  |  |  |  |  |  |  |  |
| 16466 | C | T | ORF1b | nsp13 | P77L |  |  | 13.01 | 31.85 | 7.82 | 90.65 | 98.77 | 85.89 | 100.00 | 49.76 |  |  |  |  | 58.30 |  |  |  |  |  |  |  | 89.47 | 80.85 | 99.79 | 99.56 |  |
| 16499 | A | G | ORF1b | nsp13 | Q88R |  |  |  |  |  |  |  |  | 100.00 |  |  |  |  |  |  |  |  |  |  |  |  |  |  |  |  |  |  |
| 16620 | A | T | ORF1b | nsp13 | E128D |  |  |  |  |  |  |  |  |  | 32.57 |  |  |  |  |  |  |  |  |  |  |  |  |  |  |  |  |  |
| 16650 | C | T | ORF1b | nsp13 | L138L | 100.00 | 100.00 | 100.00 | 99.59 | 99.59 | 99.58 | 100.00 | 100.00 | 83.33 |  | 99.83 | 99.89 | 98.92 | 99.69 | 99.68 | 99.91 | 99.84 | 99.88 | 99.80 | 99.89 | 99.95 | 99.80 | 99.70 | 99.81 | 99.84 | 99.76 | 99.69 |
| 16667 | C | T | ORF1b | nsp13 | T144I |  |  |  |  |  |  |  |  |  |  |  |  |  |  |  |  | 27.62 |  | 99.70 | 67.97 |  |  |  |  |  |  |  |
| 16864 | G | A | ORF1b | nsp13 | V210I |  |  |  |  |  |  |  |  |  | 75.41 |  |  |  |  |  |  |  |  |  |  |  |  |  |  |  |  |  |
| 16880 | C | T | ORF1b | nsp13 | T215I |  |  |  |  |  |  |  |  |  |  |  |  |  |  |  | 21.54 |  |  |  |  |  |  |  |  |  |  |  |
| 16917 | G | A | ORF1b | nsp13 | L227L |  |  |  |  |  |  |  |  |  |  |  |  |  |  |  |  |  |  | 99.73 |  |  |  |  |  |  |  |  |
| 17098 | A | G | ORF1b | nsp13 | K288E |  |  |  |  |  |  |  |  |  |  |  |  |  |  |  |  |  |  |  |  |  |  |  | 19.41 |  |  |  |
| 17333 | C | T | ORF1b | nsp13 | T366M |  |  |  |  |  |  |  |  |  |  |  |  |  |  |  | 22.13 |  |  |  |  | 17.29 |  |  |  |  |  |  |
| 17441 | C | A | ORF1b | nsp13 | P402H |  |  | 17.97 | 13.62 | 6.19 | 7.21 |  |  |  |  |  |  |  |  |  |  |  |  |  |  |  |  |  |  |  |  |  |
| 17456 | C | T | ORF1b | nsp13 | A407V |  |  | 4.13 | 8.88 | 51.54 |  |  |  |  |  |  |  |  |  |  |  |  |  |  |  |  |  |  |  |  |  |  |
| 17847 | G | A | ORF1b | nsp13 | Q537Q |  |  |  |  |  |  |  |  |  | 52.17 |  |  |  |  |  |  |  |  |  |  |  |  |  |  |  |  |  |
| 18027 | G | T | ORF1b | nsp13 | V597V |  |  |  |  |  |  |  |  |  |  | 99.99 | 99.99 | 100.00 | 99.97 | 100.00 | 100.00 | 99.99 | 99.99 | 99.96 | 99.99 | 99.99 | 100.00 | 99.94 | 100.00 | 100.00 | 99.97 | 100.00 |
| 18556 | A | G | ORF1b | nsp14 | T173A |  |  |  |  |  |  |  |  |  | 26.83 |  |  |  |  |  |  |  |  |  |  |  |  |  |  |  |  |  |
| 18840 | A | G | ORF1b | nsp14 | A267A |  |  |  |  |  |  |  |  |  | 22.04 |  |  |  |  |  |  |  |  |  |  |  |  |  |  |  |  |  |
| 18858 | T | C | ORF1b | nsp14 | D273D |  |  |  |  |  |  |  |  |  | 20.90 |  |  |  |  |  |  |  |  |  |  |  |  |  |  |  |  |  |
| 19052 | T | A | ORF1b | nsp14 | I338N |  |  |  |  |  |  |  |  |  | 17.99 |  |  |  |  |  |  |  |  |  |  |  |  |  |  |  |  |  |
| 19155 | A | G | ORF1b | nsp14 | T372T |  |  |  |  |  |  |  |  |  | 19.26 |  |  |  |  |  |  |  |  |  |  |  |  |  |  |  |  |  |
| 20404 | C | T | ORF1b | nsp15 | P262S |  |  |  |  |  |  |  |  |  |  |  |  |  |  |  | 21.52 |  |  |  |  |  |  |  |  |  |  |  |
| 20549 | A | G | ORF1b | nsp15 | D310G |  |  |  |  |  |  |  |  |  | 26.47 |  |  |  |  |  |  |  |  |  |  |  |  |  |  |  |  |  |
| 20675 | A | T | ORF1b | nsp16 | Q6L | 100.00 | 100.00 | 100.00 | 100.00 | 100.00 | 100.00 | 100.00 | 100.00 | 100.00 |  | 99.90 | 99.95 | 100.00 | 99.92 | 99.94 | 99.96 | 99.95 | 99.92 | 100.00 | 99.96 | 99.86 | 99.95 | 99.94 | 99.88 | 99.93 | 100.00 | 99.80 |
| 20677 | C | T | ORF1b | nsp16 | P7S |  |  |  |  |  |  |  |  | 48.98 |  |  |  |  |  |  |  |  |  |  |  |  |  |  |  |  |  |  |
| 20828 | T | C | ORF1b | nsp16 | L57S |  |  |  |  |  |  |  |  |  | 26.36 |  |  |  |  |  |  |  |  |  |  |  |  |  |  |  |  |  |

| 20904 | A | T | ORF1b | nsp16 | T82T |  |  |  |  |  |  |  |  |  | 27.47 |  |  |  |  |  |  |  |  |  |  |  |  |  |  |  |  |  |
| --- | --- | --- | --- | --- | --- | --- | --- | --- | --- | --- | --- | --- | --- | --- | --- | --- | --- | --- | --- | --- | --- | --- | --- | --- | --- | --- | --- | --- | --- | --- | --- | --- |
| 20911 | T | C | ORF1b | nsp16 | L85L |  |  |  |  |  |  |  |  |  | 28.09 |  |  |  |  |  |  |  |  |  |  |  |  |  |  |  |  |  |
| 20953 | G | A | ORF1b | nsp16 | D99N |  |  |  |  |  |  |  |  |  | 23.75 |  |  |  |  |  |  |  |  |  |  |  |  |  |  |  |  |  |
| 21005 | C | T | ORF1b | nsp16 | A116V |  |  |  |  |  |  |  |  |  |  |  |  |  |  | 55.48 |  |  |  |  |  |  |  |  | 33.61 |  |  |  |
| 21118 | T | G | ORF1b | nsp16 | C154G |  |  |  |  |  |  |  |  |  |  |  |  |  |  |  |  |  |  |  |  |  |  |  |  |  | 33.66 |  |
| 21495 | A | G | ORF1b | nsp16 | R279R |  |  |  |  |  |  |  |  |  |  |  |  |  |  |  |  |  |  |  |  |  |  |  |  |  | 19.23 |  |
| 21592 | A | G | Spike | S | L10L |  |  |  |  |  |  |  |  |  | 94.55 |  |  |  |  |  |  |  |  |  |  |  |  |  |  |  |  |  |
| 21618 | C | G | Spike | S | T19R |  |  |  |  |  |  |  |  |  | 100.00 |  |  |  |  |  |  |  |  |  |  |  |  |  |  |  |  |  |
| 21779 | A | G | Spike | S | T73A |  |  |  |  |  |  |  |  |  |  |  |  |  |  |  |  |  |  |  |  |  |  |  |  |  | 20.25 |  |
| 21991 | TTA | - | Spike | S | Y144del |  |  |  |  |  |  | 46.69 |  |  | -1.00 |  |  |  |  |  |  |  |  |  | 8.90 | 48.45 | 83.1 | 77.04 | 71.72 | 87.99 | 94.51 | 94.54 |
| 22088 | C | T | Spike | S | L176F |  |  |  |  |  |  | 18.60 |  |  | -1.00 |  |  |  |  |  |  |  |  |  |  |  |  |  |  |  |  |  |
| 22286 | C | T | Spike | S | L242F |  |  |  |  |  |  |  |  |  |  |  |  |  |  |  |  |  |  |  |  | 54.24 | 83.73 |  |  |  |  | 99.74 |
| 22599 | G | T | Spike | S | R346I |  |  |  |  |  |  |  |  |  |  |  |  |  |  |  |  |  |  |  |  |  |  |  |  | 43.83 |  |  |
| 22600 | A | T | Spike | S | R346S |  |  |  |  |  |  |  |  |  |  |  |  |  |  |  |  |  |  |  |  |  |  | 55.56 | 54.21 | 55.03 | 100.00 |  |
| 22772 | G | A | Spike | S | G404S |  |  |  |  |  |  |  |  |  | 29.68 |  |  |  |  |  |  |  |  |  |  |  |  |  |  |  |  |  |
| 23012 | G | A | Spike | S | E484K |  |  |  |  |  |  |  |  |  |  |  |  |  |  |  |  |  |  |  | 10.70 | 59.13 | 90.36 | 30.68 | 20.42 | 21.51 |  | 99.75 |
| 23013 | A | C | Spike | S | E484A |  |  |  |  |  |  |  |  |  |  |  |  |  |  |  |  |  |  |  |  |  |  | 69.22 | 42.20 | 78.52 | 100.00 |  |
| 23031 | T | C | Spike | S | F490S |  |  |  |  |  |  |  |  |  |  |  |  |  |  |  |  |  |  |  |  |  |  |  | 38.64 |  |  |  |
| 23063 | A | T | Spike | S | N501Y |  |  |  |  |  |  |  |  |  | 89.90 |  |  |  |  |  |  |  |  |  |  |  |  |  |  |  |  |  |
| 23168 | A | G | Spike | S | N536D |  |  |  |  |  |  |  |  |  | 50.86 |  |  |  |  |  |  |  |  |  |  |  |  |  |  |  |  |  |
| 23228 | A | G | Spike | S | N556D |  |  |  |  |  |  |  |  |  | 52.23 |  |  |  |  |  |  |  |  |  |  |  |  |  |  |  |  |  |
| 23271 | C | A | Spike | S | A570D |  |  |  |  |  |  |  |  |  | 88.20 |  |  |  |  |  |  |  |  |  |  |  |  |  |  |  |  |  |
| 23312 | A | G | Spike | S | I584V |  |  |  |  |  |  |  |  |  | 86.92 |  |  |  |  |  |  |  |  |  |  |  |  |  |  |  |  |  |
| 23403 | A | G | Spike | S | D614G | 100.00 | 99.51 | 100.00 | 100.00 | 99.53 | 100.00 | 100.00 | 100.00 | 100.00 | 100.00 | 99.92 | 99.87 | 98.98 | 99.88 | 99.92 | 100.00 | 99.82 | 99.85 | 99.88 | 99.96 | 99.98 | 99.90 | 100.00 | 99.91 | 99.89 | 99.83 | 99.86 |
| 23503 | A | T | Spike | S | A647A |  |  |  |  |  |  |  |  |  |  |  |  |  |  |  |  |  |  |  |  | 76.74 | 70.21 |  |  |  |  | 99.94 |
| 23604 | C | G | Spike | S | P681R |  |  |  |  |  |  |  |  |  | 100.00 |  |  |  |  |  |  |  |  |  |  |  |  |  |  |  |  |  |
| 23709 | C | T | Spike | S | T716I |  |  |  |  |  |  |  |  | 89.29 |  |  |  |  |  |  |  |  |  |  |  |  |  |  |  |  |  |  |
| 23764 | A | G | Spike | S | T734T |  |  |  |  |  |  |  |  |  | 34.29 |  |  |  |  |  |  |  |  |  |  |  |  |  |  |  |  |  |
| 24066 | A | G | Spike | S | K835R |  |  |  |  |  |  |  |  |  |  |  |  |  |  |  |  |  |  |  |  |  |  |  |  |  | 35.03 |  |
| 24410 | G | A | Spike | S | D950N |  |  |  |  |  |  |  |  |  | 99.57 |  |  |  |  |  |  |  |  |  |  |  |  |  |  |  |  |  |
| 24509 | C | T | Spike | S | R983C |  |  |  |  |  |  |  |  |  |  |  |  |  |  |  |  |  |  |  |  |  |  | 14.60 |  |  |  |  |
| 24695 | A | G | Spike | S | K1045E |  |  |  |  |  |  |  |  |  | 19.46 |  |  |  |  |  |  |  |  |  |  |  |  |  |  |  |  |  |
| 24795 | C | T | Spike | S | A1078V |  |  |  |  |  |  | 57.72 |  |  |  |  |  |  |  |  |  |  |  |  |  |  |  |  |  |  |  |  |
| 24820 | A | G | Spike | S | K1086K |  |  |  |  |  |  |  |  |  | 19.35 |  |  |  |  |  |  |  |  |  |  |  |  |  |  |  |  |  |
| 24859 | C | T | Spike | S | G1099G |  |  |  |  |  |  |  |  |  |  |  |  |  |  | 55.32 |  |  |  |  |  |  |  |  | 42.79 |  |  |  |
| 24977 | G | C | Spike | S | D1139H |  |  |  |  |  | 44.63 |  |  |  |  |  |  |  |  |  |  |  |  |  |  |  |  |  |  |  |  |  |
| 25049 | G | T | Spike | S | D1163Y |  |  |  |  |  |  |  |  |  |  |  |  |  |  |  |  |  |  |  |  |  |  |  | 20.12 |  |  |  |
| 25291 | T | C | Spike | S | C1243C |  |  |  |  |  |  |  |  |  | 39.22 |  |  |  |  |  |  |  |  |  |  |  |  |  |  |  |  |  |
| 25413 | C | T | ORF3a | ORF3a | I7I |  |  |  |  |  |  |  |  |  |  |  |  |  |  |  | 21.17 |  |  |  |  |  |  |  |  |  |  |  |
| 25469 | C | T | ORF3a | ORF3a | S26L |  |  |  |  |  |  |  |  |  | 87.83 |  |  |  |  |  |  |  |  |  |  |  |  |  |  |  |  |  |
| 25563 | G | T | ORF3a | ORF3a | Q57H | 100.00 | 100.00 | 100.00 | 100.00 | 99.58 | 100.00 | 100.00 | 100.00 | 100.00 |  | 99.97 | 99.96 | 99.95 | 100.00 | 100.00 | 99.97 | 99.97 | 99.93 | 99.92 | 99.97 | 99.97 | 99.99 | 99.78 | 100.00 | 99.91 | 100.00 | 99.91 |
| 25649 | T | C | ORF3a | ORF3a | L86S |  |  |  |  |  |  |  |  |  | 18.18 |  |  |  |  |  |  |  |  |  |  |  |  |  |  |  |  |  |
| 25658 | C | T | ORF3a | ORF3a | T89I |  |  |  |  |  | 91.60 |  |  |  |  |  |  |  |  |  |  |  |  |  |  |  |  |  |  |  |  |  |
| 25720 | G | T | ORF3a | ORF3a | A110S |  |  |  |  |  |  |  |  |  | 80.16 |  |  |  |  |  |  |  |  |  |  |  |  |  |  |  |  |  |
| 25912 | G | T | ORF3a | ORF3a | G174C |  |  |  |  |  |  |  |  |  |  | 99.99 | 99.99 | 100.00 | 99.78 | 100.00 | 99.95 | 89.83 | 99.97 | 100.00 | 99.99 | 99.97 | 99.91 | 100.00 | 100.00 | 99.85 | 100.00 | 99.90 |
| 26491 | T | C | M | M | upstream |  |  |  |  |  |  |  |  |  |  |  |  |  |  |  |  |  |  |  |  |  |  |  |  |  | 62.03 |  |
| 26492 | A | T | M | M | upstream |  |  |  |  |  |  |  |  |  |  |  |  |  |  |  |  |  |  |  |  |  |  |  |  |  | 62.07 |  |
| 26681 | C | T | M | M | F53F |  |  |  |  |  |  |  |  |  |  |  |  |  |  |  |  |  |  | 18.02 |  |  |  |  |  |  |  |  |
| 26767 | T | C | M | M | I82T |  |  |  |  |  |  |  |  |  | 31.82 |  |  |  |  |  |  |  |  |  |  |  |  |  |  |  |  |  |
| 26801 | C | T | M | M | L93L |  |  |  |  |  |  |  | 27.68 |  |  |  |  |  |  |  |  |  |  | 19.95 |  |  |  |  |  |  |  |  |
| 27559 | C | T | ORF7a | ORF7a | L56L |  |  |  |  |  |  |  |  | 10.00 | 82.43 |  |  |  |  |  |  |  |  |  |  |  |  |  |  |  |  |  |
| 27638 | T | C | ORF7a | ORF7a | V82A |  |  |  |  |  |  |  |  | 9.09 | 86.81 |  |  |  |  |  |  |  |  |  |  |  |  |  |  |  |  |  |
| 27739 | C | T | ORF7a | ORF7a | L116F |  |  |  |  |  |  |  |  |  | 17.95 |  |  |  |  |  |  |  |  |  |  |  |  |  |  |  |  |  |
| 27752 | C | T | ORF7a | ORF7a | T120I |  |  |  |  |  |  |  |  | 9.58 | 78.03 |  |  |  |  |  |  |  |  |  |  |  |  |  |  |  |  |  |
| 27906 | G | A | ORF8 | ORF8 | V5I |  |  |  |  |  |  |  |  |  |  |  |  |  |  |  |  |  |  |  |  |  | 27.52 |  |  |  |  |  |
| 27972 | C | T | ORF8 | ORF8 | Q27* |  |  |  |  |  |  |  |  |  | 46.06 |  |  |  |  |  |  |  |  |  |  |  |  |  |  |  |  |  |
| 28048 | G | T | ORF8 | ORF8 | R52I |  |  |  |  |  |  |  |  |  | 44.53 |  |  |  |  |  |  |  |  |  |  |  |  |  |  |  |  |  |
| 28090 | G | T | ORF8 | ORF8 | G66V |  |  |  |  |  |  |  |  |  | 53.56 |  |  |  |  |  |  |  |  |  |  |  |  |  |  |  |  |  |
| 28095 | A | T | ORF8 | ORF8 | K68* |  |  |  |  |  |  |  |  |  | 45.19 |  |  |  |  |  |  |  |  |  |  |  |  |  |  |  |  |  |
| 28111 | A | G | ORF8 | ORF8 | Y73C |  |  |  |  |  |  |  |  |  | 42.26 |  |  |  |  |  |  |  |  |  |  |  |  |  |  |  |  |  |
| 28247 | AGATTTC | A | ORF8 | ORF8 | D119_F120del |  |  |  |  |  |  |  |  |  | 61.34 |  |  |  |  |  |  |  |  |  |  |  |  |  |  |  |  |  |
| 28270 | TAAAA | TAAA | N | N | upstream |  |  |  |  |  |  |  | 11.79 |  | 100.00 |  |  |  |  |  |  |  |  |  |  |  |  |  |  |  |  |  |
| 28271 | A | T | N | N | upstream |  |  |  |  |  |  |  |  |  |  |  |  |  |  |  |  |  |  | 17.39 |  |  |  |  |  |  |  |  |
| 28271 | A | G | N | N | upstream |  |  |  |  |  |  |  | 58.14 |  |  |  |  |  |  |  |  |  |  |  |  |  |  |  |  |  |  |  |
| 28280 | G | C | N | N | D3H |  |  |  |  |  |  |  |  |  | 36.14 |  |  |  |  |  |  |  |  |  |  |  |  |  |  |  |  |  |
| 28281 | A | T | N | N | D3V |  |  |  |  |  |  |  |  |  | 36.00 |  |  |  |  |  |  |  |  |  |  |  |  |  |  |  |  |  |
| 28282 | T | A | N | N | D3E |  |  |  |  |  |  |  |  |  | 36.14 |  |  |  |  |  |  |  |  |  |  |  |  |  |  |  |  |  |
| 28461 | A | G | N | N | D63G |  |  |  |  |  |  |  |  |  | 54.73 |  |  |  |  |  |  |  |  |  |  |  |  |  |  |  |  |  |
| 28606 | C | T | N | N | Y111Y |  |  |  |  |  |  |  |  |  |  | 99.72 | 99.81 | 99.66 | 99.87 | 99.66 | 99.79 | 99.75 | 99.78 | 99.76 | 99.76 | 99.83 | 99.76 | 99.67 | 99.56 | 99.70 | 99.38 | 99.45 |
| 28881 | GGGG | AACC | N | N | R203_G204delinsKR |  |  |  |  |  |  |  |  |  | 87.00 |  |  |  |  |  |  |  |  |  |  |  |  |  |  |  |  |  |
| 28922 | G | T | N | N | A217S |  |  | 28.46 | 10.48 |  | 7.72 |  |  |  |  |  |  |  |  |  |  |  |  |  |  |  |  |  |  |  |  |  |
| 28948 | C | T | N | N | D225D |  |  |  |  |  |  |  |  | 21.24 |  |  |  |  |  |  |  |  |  |  |  |  |  |  |  |  |  |  |
| 28977 | C | T | N | N | S235F |  |  |  |  |  |  |  |  |  | 74.90 |  |  |  |  |  |  |  |  |  |  |  |  |  |  |  |  |  |
| 29144 | C | T | N | N | L291L |  |  |  |  |  |  |  |  |  | 20.61 |  |  |  |  |  |  |  |  |  |  |  |  |  |  |  |  |  |
| 29179 | G | T | N | N | P302P | 100.00 | 100.00 | 100.00 | 100.00 | 100.00 | 100.00 | 100.00 | 100.00 | 100.00 |  | 99.97 | 99.95 | 100.00 | 99.96 | 100.00 | 99.96 | 99.89 | 99.95 | 99.82 | 99.97 | 99.92 | 99.92 | 99.82 | 99.91 | 99.84 | 99.50 | 99.76 |
| 29316 | A | G | N | N | D348G |  |  |  |  |  |  |  |  |  | 16.96 |  |  |  |  |  |  |  |  |  |  |  |  |  |  |  |  |  |
| 29336 | A | G | N | N | K355E |  |  |  |  |  |  |  |  |  | 19.38 |  |  |  |  |  |  |  |  |  |  |  |  |  |  |  |  |  |
| 29402 | G | T | N | N | D377Y |  |  |  |  |  |  |  |  | 11.93 | 64.29 |  |  |  |  |  |  |  |  |  |  |  |  |  |  |  |  |  |
| 29520 | C | T | N | N | S416L |  |  |  |  |  |  |  |  |  | 19.92 |  |  |  |  |  |  |  |  |  |  |  |  |  |  |  |  |  |
| 29625 | C | T | ORF10 | ORF10 | S23F |  |  |  |  |  |  |  |  |  |  | 99.73 | 99.68 | 99.56 | 99.69 | 99.69 | 99.81 | 99.74 | 99.66 | 99.57 | 99.79 | 99.76 | 99.72 | 99.67 | 99.86 | 99.68 | -1.00 | 100.00 |
| 29739 | C | T |  | 3'UTR |  |  |  |  |  |  |  |  |  |  |  |  |  |  |  |  |  |  |  |  |  |  |  | 47.96 |  |  |  |  |
| 29742 | G | T |  | 3'UTR |  |  |  |  |  |  |  |  |  | 15.38 | 78.48 |  |  |  |  |  |  |  |  |  |  |  |  |  |  |  |  |  |
| 29745 | G | T |  | 3'UTR |  |  |  |  |  |  |  |  |  |  |  | 100.00 | 100.00 | 99.92 | 99.97 | 99.92 | 100.00 | 100.00 | 100.00 | 100.00 | 99.80 | 99.90 | 100.00 | 100.00 | 100.00 | 100.00 | -1.00 | 100.00 |
| 29755 | G | T |  | 3'UTR |  |  |  |  |  |  |  |  |  |  |  | 100.00 | 100.00 | 100.00 | 99.96 | 100.00 | 100.00 | 99.82 | 100.00 | 99.93 | 100.00 | 100.00 | 100.00 | 100.00 | 99.90 | 99.65 | -1.00 | 100.00 |
