# Supplementary material for: SARS-CoV-2 shedding dynamics and transmission in immunosuppressed patients
Source: Virulence. 2022 Jul 26;13(1):1242–51. doi: 10.1080/21505594.2022.2101198 (PMC9336477; doi:10.1080/21505594.2022.2101198)
Supplement: Supplemental Material [file KVIR_A_2101198_SM8377.zip › supplementary/4. R4_Supplementary File_covid19_dynamics_Virulence_220630.docx]

SARS-CoV-2 shedding dynamics and transmission in immunosuppressed patients

Supplementary Data

Table of Contents

**Supplementary Figures**

Figure S1. Distribution of collection date range of total dataset and subsampled dataset…………2

Figure S2. Time-resolved phylogenetic analysis of severe acute respiratory syndrome coronavirus 2 strains…….3

Figure S3. Variant distribution per month in all complete sequences sourced from Korea in the GISAID database (https://www.gisaid.org/). The asterisk symbol (*) represents collection of day 156 sample (containing N501Y and P681R) from patient 1……………………………………….4

Figure S4. Short tandem repeat (STR) analysis of genomic DNA obtained respiratory samples from patient 1………………………………………………………………………………………5

Figure S5. Integrative Genomic Viewer (IGV) window snapshot showing aligned sequencing reads of SARS-CoV-2 from patient 2 on day 59. 6

Figure S6. Integrative Genomic Viewer (IGV) window snapshot showing aligned sequencing reads of SARS-CoV-2 from patient 2 on day 63.

**Supplementary Tables**

Table S1. Primers for whole genome sequencing of SARS-CoV-2………………………………….7

Table S2. Accession numbers for the 149 genomes obtained from GISAID 8

Table S3. Clinical characteristics of patients analyzed in this study 9

Table S4. Statistics of whole genome sequencing performed in this study 10

Table S5. SARS-CoV-2 variant and allele frequency changes in clinical samples……………………11

Table S6. Transmission bottle size estimated using a beta-binomial model ..12

**Figure S1. Distribution of collection date range of total dataset and subsampled dataset.**

**
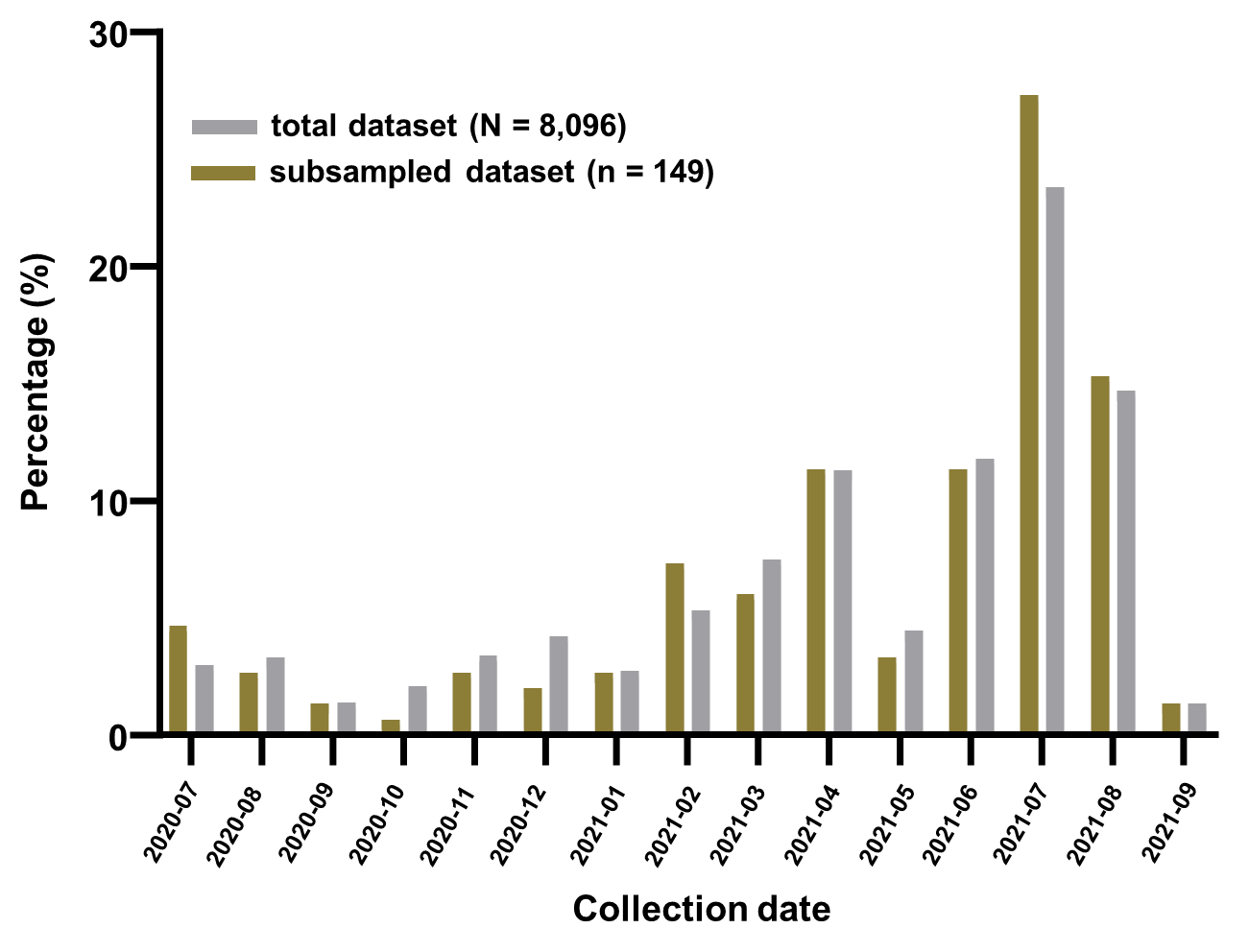
**

**Figure S2. Time-resolved phylogenetic analysis of severe acute respiratory syndrome coronavirus 2 strains.**

**
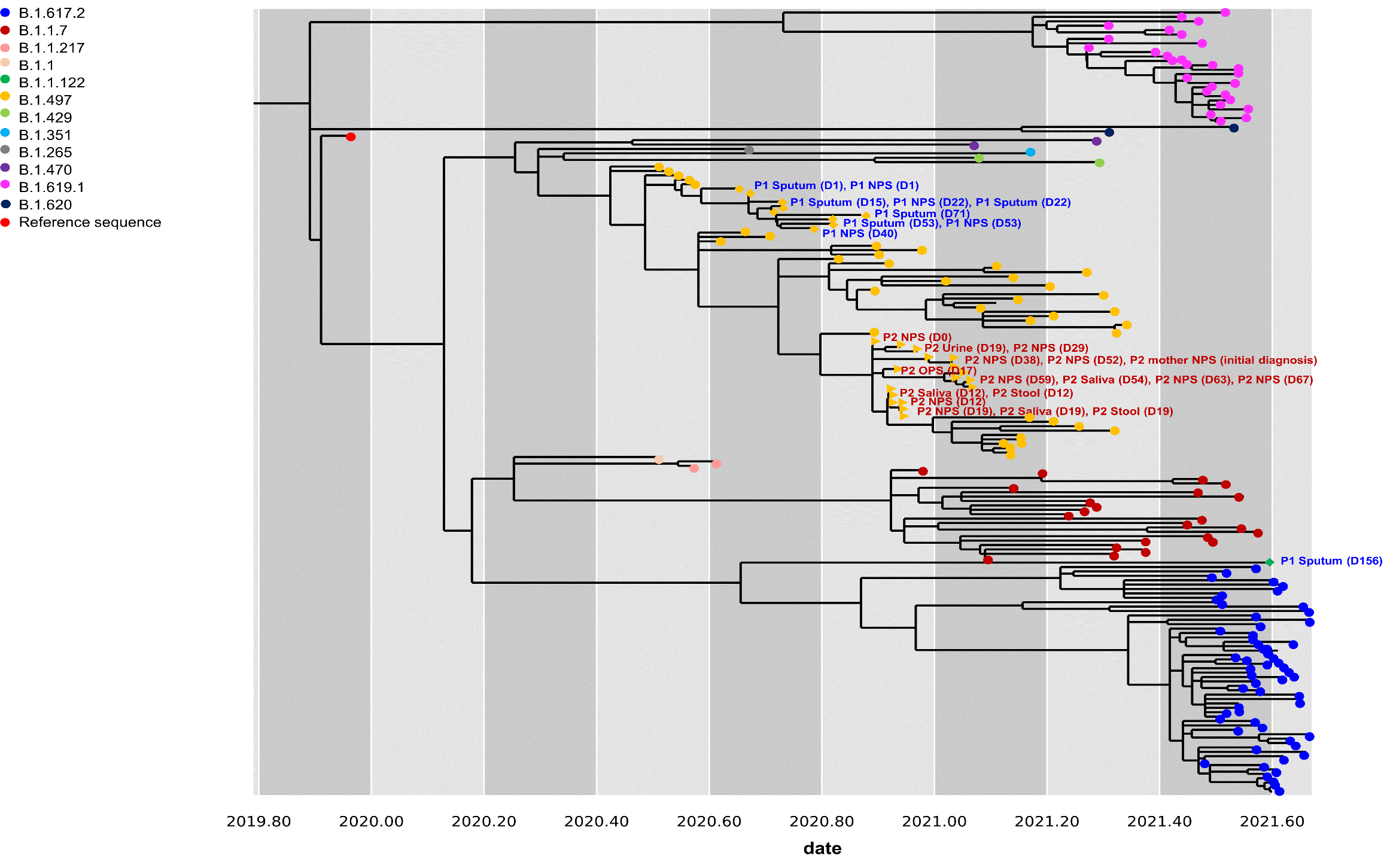
**

**Figure S3. Variant distribution per month in all complete sequences sourced from Korea in the GISAID database (**[**https://www.gisaid.org/**](https://www.gisaid.org/)**). The asterisk symbol (*) represents collection of day 156 sample (containing N501Y and P681R) from patient 1.**


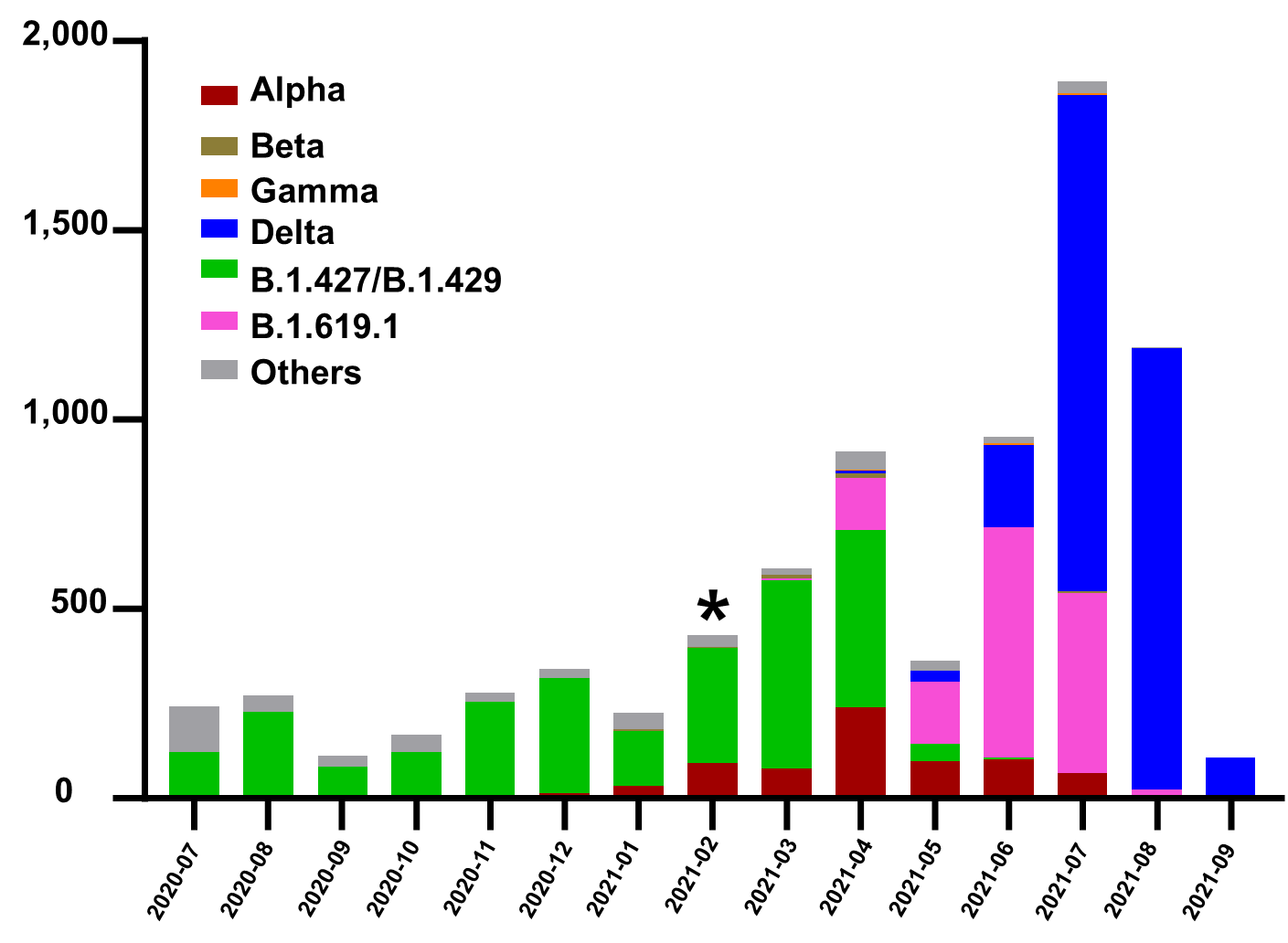


**Figure S4. Short tandem repeat (STR) analysis of genomic DNA obtained respiratory samples from patient 1.**


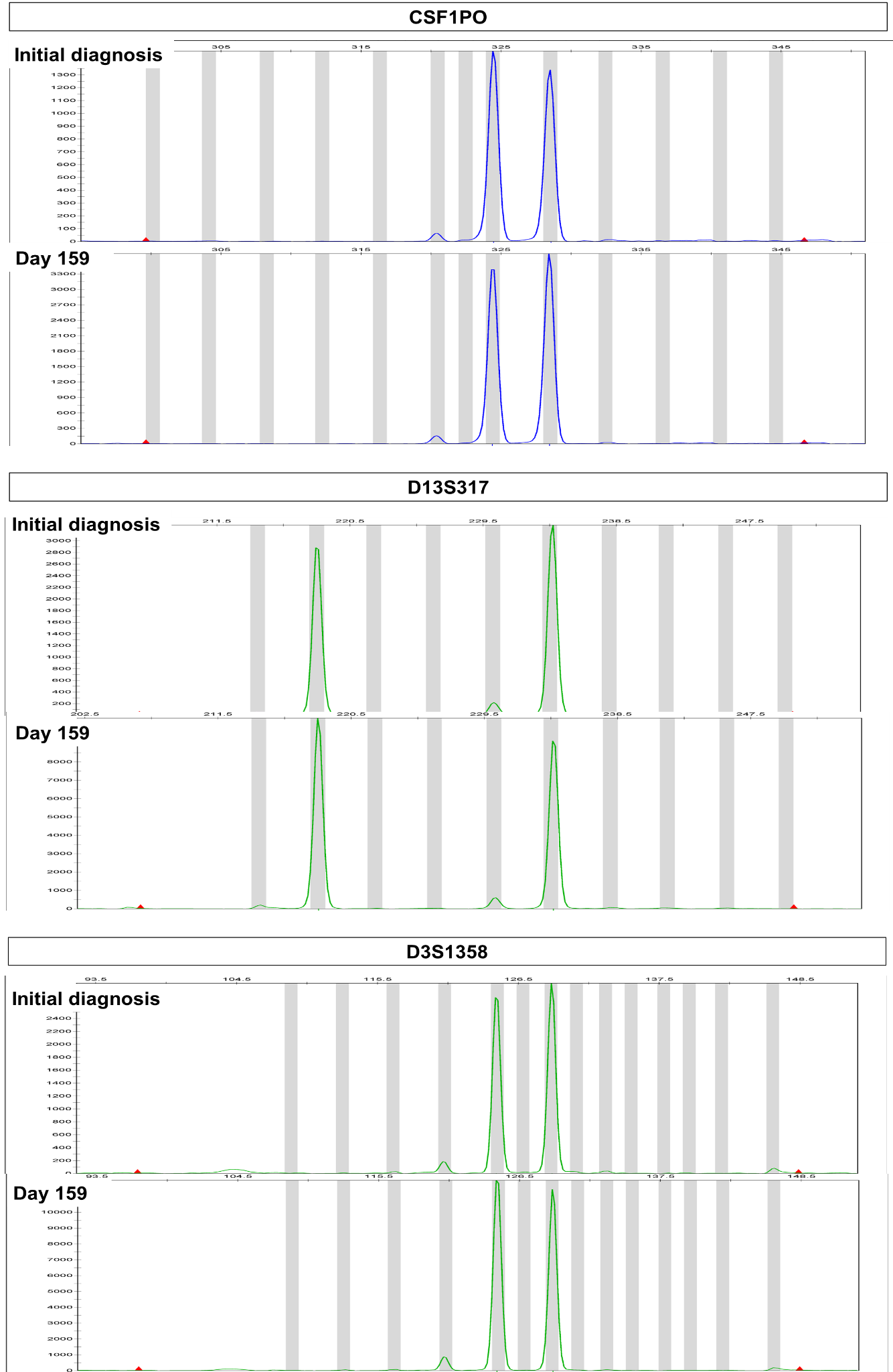


Figure S5. Integrative Genomic Viewer (IGV) window snapshot showing aligned sequencing reads of SARS-CoV-2 from patient 2 on day 59.


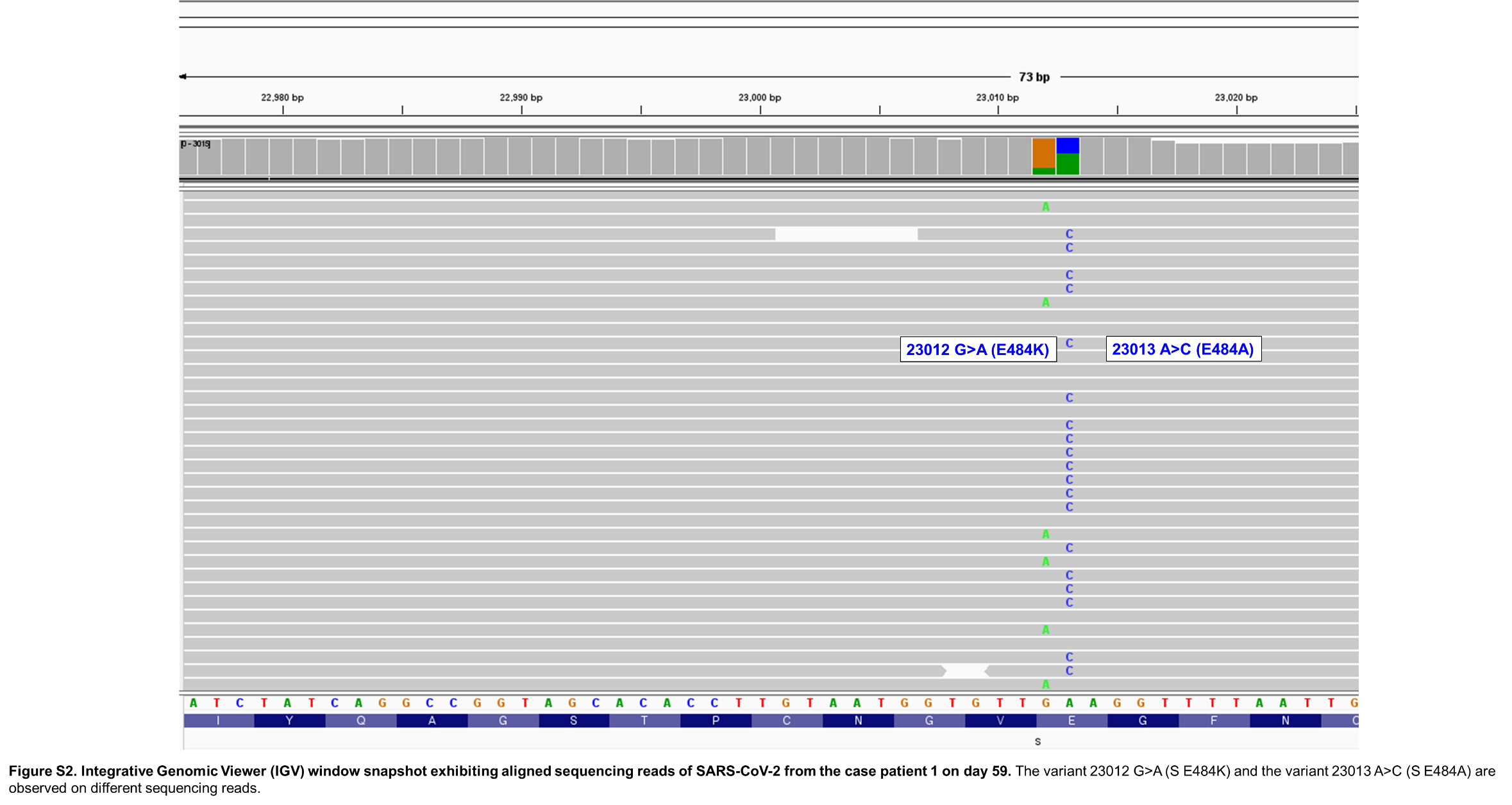


Figure S6. Integrative Genomic Viewer (IGV) window snapshot showing aligned sequencing reads of SARS-CoV-2 from patient 2 on day 63.


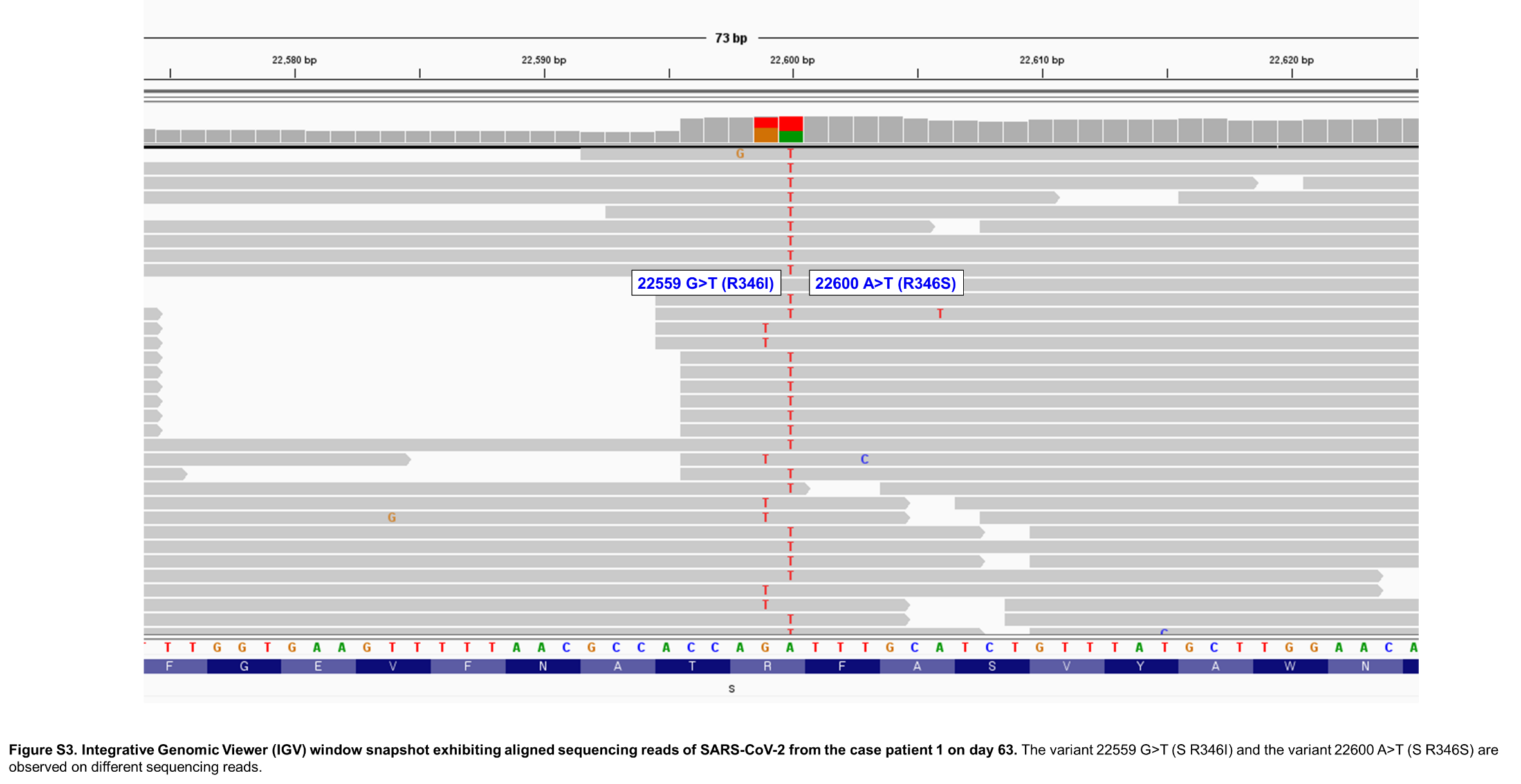


Table S1. Primers for whole genome sequencing of SARS-CoV-2

| **Primer** | **Position** | **Nucleotide sequence (Forward, 5' - 3')** | **Nucleotide sequence (Reverse, 5' - 3')** |
| --- | --- | --- | --- |
| nCOV_new_F1 | 15-815 | CCTTCCCAGGTAACAAACCA | GAGTGTATGCCCCTCCGTTA |
| nCOV_new_F2 | 746-1727 | TGGAACACTAAACATAGCAGTGG | CAAAAGCACTTGTGGAAGCA |
| nCOV_new_F3 | 1464-2460 | GTGGTCGCACTATTGCCTTT | GGCATGAGTAGGCCAGTTTC |
| nCOV_new_F4 | 2405-3004 | AAGGGATTGTACAGAAAGTGTGTT | CTCACCAGACTCATCAAATAAGTAGT |
| nCOV_new_F5-1 | 2763-3450 | TGCAAGGTTACAAGAGTGTGAA | TTGGCTGCATTAACAACCAC |
| nCOV_new_F5-2 | 3238-3737 | AGACGGCAGTGAGGACAATC | GGTCAGCACCAAAAATACCAG |
| nCOV_new_F6 | 3578-4525 | TTAAGCGGACACAATCTTGC | AGCACCATAATCAACCACACC |
| nCOV_new_F7 | 4254-5212 | CGGGTCAGGGTTTAAATGGT | TGCTGACATGTACCTACCCAGA |
| nCOV_new_F8 | 5003-6002 | CACACGCAAGTTGTGGACAT | CAATTGGTTGCTCTGTGAAA |
| 3_1 | 5934-6851 | CAGAAATTGACCCTAAGTTGGA | TCGGCATAGATGCTTTAATTC |
| 3_2 | 6740-7684 | CGGTGTTTAAACCGTGTTTGT | CAAGTCTCTCGCAACTTCATCA |
| 3_3 | 7456-8444 | TGTGCATGTTGTAGACGGTTG | TTCGTAGTTGTTCAGACAATGACA |
| 3_4 | 8375-9224 | GCAAAAAGTCACAACATTGCTT | CGTGCCTACAGTACTCAGAATCA |
| 3_5 | 9127-10033 | TGTGCTCATGGATGGCTCTA | AGAGGTTTGTGGTGGTTGGT |
| 3_6 | 9956-10827 | TGTTGTCATCTCGCAAAGGC | CCAGTTTGAGCAGAAAGAGGT |
| 3_7 | 10771-11768 | TGAACCTCTAACACAAGACCATG | TGCTATTCTTGGGTGGGAGT |
| 3_8 | 11547-12470 | TGTGTGTTGAGTATTGCCCT | TGGCTGCTGTTGTAAGAGGT |
| nCOV_new_F9 | 12212-13210 | TCTTTGAATGTGGCTAAATCTGA | ATTGGCTTCCGGTGTAACTG |
| nCOV_new_F10-1 | 13004-13497 | GCTGCCACAGTACGTCTACAA | AAGACGGGCTGCACTTACAC |
| nCOV_new_F10-2 | 13327-13823 | AACTTGTGCTAATGACCCTGTG | ACGAGGTCTGCCATTGTGTA |
| nCOV_new_F11 | 13663-14639 | CACACTTTCTCTAACTACCAACATGAA | GCAGCTACTGAAAAGCACGTA |
| nCOV_new_F12 | 14401-15392 | TTCCCACCTACAAGTTTTGGA | CGGTGTGACAAGCTACAACA |
| nCOV_new_F13 | 15167-16139 | TGAAATCAATAGCCGCCACT | AACATGTGTCCTGTTAACTCATCA |
| nCOV_new_F14 | 15862-16861 | GGACCTCATGAATTTTGCTCTC | CAGCATCACCATAGTCACCTTT |
| nCOV_new_F15 | 16573-17571 | GACTGGACAAATGCTGGTGA | AGGACAACGCCGACAAGTT |
| nCOV_new_F16 | 17477-18176 | AGGGCACACTAGAACCAGAA | GGTATGCCAGGTATGTCAACAC |
| nCOV_new_F17 | 18103-19049 | CAGGCACCTACACACCTCAG | GCTTTAGGGTTACCAATGTCG |
| nCOV_new_F18 | 18765-19738 | TGATGTTCAACAATGGGGTTT | CATCAACACCATCAACTTTTGTG |
| nCOV_new_F19 | 19506-20478 | TGAGTACAGATTGTATCTCGATGCT | ACCTGTTTGCGCATCTGTTA |
| nCOV_new_F20 | 20232-21230 | ACCCAGGAGTCAAATGGAAA | GTCCACCATGCGAAGTGTC |
| 8_1 | 20914-21912 | AAGACAGTGGTTGCCTACGG | GGGACTGGGTCTTCGAATCT |
| 8_2 | 21776-22623 | TGGGACCAATGGTACTAAGAGG | TCCAAGCATAAACAGATGCAA |
| 8_3 | 22580-23429 | TGAAGTTTTTAACGCCACCA | AACAGGGACTTCTGTGCAGTT |
| 8_4 | 23375-24224 | TTCTAACCAGGTTGCTGTTCTTT | GGTCCAACCAGAAGTGATTGT |
| nCOV_new_F21 | 23893-24885 | CACCCAAGAAGTTTTTGCAC | TTCCTTTGTGTTACAAACCAGTG |
| nCOV_new_F22 | 24685-25684 | TTTTTGTGGAAAGGGCTATCA | CAACGAGCAAAAGGTGTGAG |
| nCOV_new_F23 | 25436-26380 | TGAAGCAAGGTGAAATCAAGG | TATTGCAGCAGTACGCACAC |
| nCOV_new_F24 | 26181-27017 | TTATGATGAACCGACGACGA | AGGCAGGTCCTTGATGTCAC |
| nCOV_new_F25 | 26936-27897 | CGTAATCGGAGCTGTGATCC | TCATGTTCGTTTAGGCGTGA |
| nCOV_new_F26 | 27701-28693 | TTGTTGCGGCAATAGTGTTT | ATTCAAGGCTCCCTCAGTTG |
| nCOV_new_F27 | 28445-29426 | CAACATGGCAAGGAAGACCT | TCTGCGGTAAGGCTTGAGTT |
| nCOV_new_F28 | 29356-29787 | AACATTCCCACCAACAGAGC | GCAGCTCTCCCTAGCATTGT |

Table S2. Accession numbers for the 149 genomes obtained from GISAID^1^

| **Accession ID** | **Isolate name** | **Location** | **Collection date** |
| --- | --- | --- | --- |
| EPI_ISL_522472 | hCoV-19/SouthKorea/KCDC2692/2020 | South Korea | 2020-07-02 |
| EPI_ISL_1063653 | hCoV-19/SouthKorea/KDCA1344/2020 | South Korea | 2020-07-05 |
| EPI_ISL_515038 | hCoV-19/SouthKorea/KCDC2636/2020 | South Korea | 2020-07-07 |
| EPI_ISL_522517 | hCoV-19/SouthKorea/KCDC2738/2020 | South Korea | 2020-07-16 |
| EPI_ISL_526701 | hCoV-19/SouthKorea/KCDC2767/2020 | South Korea | 2020-07-22 |
| EPI_ISL_526721 | hCoV-19/SouthKorea/KCDC2787/2020 | South Korea | 2020-07-24 |
| EPI_ISL_526725 | hCoV-19/SouthKorea/KCDC2791/2020 | South Korea | 2020-07-25 |
| EPI_ISL_850292 | hCoV-19/SouthKorea/KDCA0608/2020 | South Korea | 2020-08-10 |
| EPI_ISL_1490177 | hCoV-19/SouthKorea/KDCA2720/2020 | South Korea | 2020-08-12 |
| EPI_ISL_850456 | hCoV-19/SouthKorea/KDCA0779/2020 | South Korea | 2020-08-25 |
| EPI_ISL_850467 | hCoV-19/SouthKorea/KDCA0790/2020 | South Korea | 2020-08-27 |
| EPI_ISL_1063667 | hCoV-19/SouthKorea/KDCA1385/2020 | South Korea | 2020-09-01 |
| EPI_ISL_747291 | hCoV-19/SouthKorea/KDCA0050/2020 | South Korea | 2020-09-15 |
| EPI_ISL_760177 | hCoV-19/SouthKorea/KDCA0393/2020 | South Korea | 2020-10-31 |
| EPI_ISL_747401 | hCoV-19/SouthKorea/KDCA0167/2020 | South Korea | 2020-11-19 |
| EPI_ISL_955939 | hCoV-19/SouthKorea/KDCA0841/2020 | South Korea | 2020-11-19 |
| EPI_ISL_747458 | hCoV-19/SouthKorea/KDCA0225/2020 | South Korea | 2020-11-23 |
| EPI_ISL_959500 | hCoV-19/SouthKorea/KDCA0924/2020 | South Korea | 2020-11-25 |
| EPI_ISL_994733 | hCoV-19/SouthKorea/KDCA1017/2020 | South Korea | 2020-12-03 |
| EPI_ISL_2161145 | hCoV-19/SouthKorea/KDCA3659/2020 | South Korea | 2020-12-23 |
| EPI_ISL_850654 | hCoV-19/SouthKorea/KDCA0830/2020 | South Korea | 2020-12-24 |
| EPI_ISL_995772 | hCoV-19/SouthKorea/KDCA1208/2021 | South Korea | 2021-01-09 |
| EPI_ISL_1315339 | hCoV-19/SouthKorea/KDCA2047/2021 | South Korea | 2021-01-25 |
| EPI_ISL_1063721 | hCoV-19/SouthKorea/KDCA1535/2021 | South Korea | 2021-01-29 |
| EPI_ISL_1209427 | hCoV-19/SouthKorea/KDCA1810/2021 | South Korea | 2021-01-31 |
| EPI_ISL_1165010 | hCoV-19/SouthKorea/KDCA1788/2021 | South Korea | 2021-02-03 |
| EPI_ISL_1138951 | hCoV-19/SouthKorea/KDCA1683/2021 | South Korea | 2021-02-08 |
| EPI_ISL_1138954 | hCoV-19/SouthKorea/KDCA1686/2021 | South Korea | 2021-02-11 |
| EPI_ISL_1228617 | hCoV-19/SouthKorea/KDCA1890/2021 | South Korea | 2021-02-14 |
| EPI_ISL_1315398 | hCoV-19/SouthKorea/KDCA2110/2021 | South Korea | 2021-02-17 |
| EPI_ISL_1315410 | hCoV-19/SouthKorea/KDCA2122/2021 | South Korea | 2021-02-17 |
| EPI_ISL_1489565 | hCoV-19/SouthKorea/KDCA2232/2021 | South Korea | 2021-02-19 |
| EPI_ISL_1165063 | hCoV-19/SouthKorea/KDCA1779/2021 | South Korea | 2021-02-20 |
| EPI_ISL_1252440 | hCoV-19/SouthKorea/KDCA2028/2021 | South Korea | 2021-02-20 |
| EPI_ISL_1228637 | hCoV-19/SouthKorea/KDCA1913/2021 | South Korea | 2021-02-23 |
| EPI_ISL_1315360 | hCoV-19/SouthKorea/KDCA2068/2021 | South Korea | 2021-02-26 |
| EPI_ISL_1622488 | hCoV-19/SouthKorea/KDCA2926/2021 | South Korea | 2021-03-01 |
| EPI_ISL_1489642 | hCoV-19/SouthKorea/KDCA2330/2021 | South Korea | 2021-03-02 |
| EPI_ISL_1489626 | hCoV-19/SouthKorea/KDCA2305/2021 | South Korea | 2021-03-03 |
| EPI_ISL_1622337 | hCoV-19/SouthKorea/KDCA2853/2021 | South Korea | 2021-03-07 |
| EPI_ISL_1490071 | hCoV-19/SouthKorea/KDCA2561/2021 | South Korea | 2021-03-16 |
| EPI_ISL_1489982 | hCoV-19/SouthKorea/KDCA2404/2021 | South Korea | 2021-03-17 |
| EPI_ISL_1489989 | hCoV-19/SouthKorea/KDCA2418/2021 | South Korea | 2021-03-17 |
| EPI_ISL_2332277 | hCoV-19/SouthKorea/KDCA3922/2021 | South Korea | 2021-03-26 |
| EPI_ISL_1675282 | hCoV-19/SouthKorea/KDCA2986/2021 | South Korea | 2021-04-05 |
| EPI_ISL_1934688 | hCoV-19/SouthKorea/KDCA3047/2021 | South Korea | 2021-04-06 |
| EPI_ISL_2332423 | hCoV-19/SouthKorea/KDCA4113/2021 | South Korea | 2021-04-09 |
| EPI_ISL_2361117 | hCoV-19/SouthKorea/KDCA4345/2021 | South Korea | 2021-04-09 |
| EPI_ISL_2332323 | hCoV-19/SouthKorea/KDCA3980/2021 | South Korea | 2021-04-10 |

| EPI_ISL_1934845 | hCoV-19/SouthKorea/KDCA3237/2021 | South Korea | 2021-04-13 |
| --- | --- | --- | --- |
| EPI_ISL_1934853 | hCoV-19/SouthKorea/KDCA3245/2021 | South Korea | 2021-04-13 |
| EPI_ISL_2332342 | hCoV-19/SouthKorea/KDCA4006/2021 | South Korea | 2021-04-14 |
| EPI_ISL_1936628 | hCoV-19/SouthKorea/KDCA3449/2021 | South Korea | 2021-04-17 |
| EPI_ISL_2361174 | hCoV-19/SouthKorea/KDCA4395/2021 | South Korea | 2021-04-21 |
| EPI_ISL_2754341 | hCoV-19/SouthKorea/KDCA4846/2021 | South Korea | 2021-04-22 |
| EPI_ISL_2361207 | hCoV-19/SouthKorea/KDCA4454/2021 | South Korea | 2021-04-23 |
| EPI_ISL_2332407 | hCoV-19/SouthKorea/KDCA4088/2021 | South Korea | 2021-04-24 |
| EPI_ISL_1936665 | hCoV-19/SouthKorea/KDCA3487/2021 | South Korea | 2021-04-26 |
| EPI_ISL_2464351 | hCoV-19/SouthKorea/KDCA4601/2021 | South Korea | 2021-04-26 |
| EPI_ISL_1936672 | hCoV-19/SouthKorea/KDCA3499/2021 | South Korea | 2021-04-27 |
| EPI_ISL_2284651 | hCoV-19/SouthKorea/KDCA3834/2021 | South Korea | 2021-04-27 |
| EPI_ISL_2284596 | hCoV-19/SouthKorea/KDCA3771/2021 | South Korea | 2021-05-01 |
| EPI_ISL_2332570 | hCoV-19/SouthKorea/KDCA4307/2021 | South Korea | 2021-05-12 |
| EPI_ISL_2332566 | hCoV-19/SouthKorea/KDCA4299/2021 | South Korea | 2021-05-13 |
| EPI_ISL_2754391 | hCoV-19/SouthKorea/KDCA4900/2021 | South Korea | 2021-05-21 |
| EPI_ISL_2754438 | hCoV-19/SouthKorea/KDCA4952/2021 | South Korea | 2021-05-27 |
| EPI_ISL_2754511 | hCoV-19/SouthKorea/KDCA5042/2021 | South Korea | 2021-06-01 |
| EPI_ISL_2754657 | hCoV-19/SouthKorea/KDCA5201/2021 | South Korea | 2021-06-07 |
| EPI_ISL_2754661 | hCoV-19/SouthKorea/KDCA5205/2021 | South Korea | 2021-06-07 |
| EPI_ISL_2754713 | hCoV-19/SouthKorea/KDCA5263/2021 | South Korea | 2021-06-09 |
| EPI_ISL_2754788 | hCoV-19/SouthKorea/KDCA5369/2021 | South Korea | 2021-06-14 |
| EPI_ISL_3933498 | hCoV-19/SouthKorea/KDCA10515/2021 | South Korea | 2021-06-14 |
| EPI_ISL_2967332 | hCoV-19/SouthKorea/KDCA5611/2021 | South Korea | 2021-06-19 |
| EPI_ISL_3026111 | hCoV-19/SouthKorea/KDCA5955/2021 | South Korea | 2021-06-21 |
| EPI_ISL_2967426 | hCoV-19/SouthKorea/KDCA5712/2021 | South Korea | 2021-06-22 |
| EPI_ISL_3026113 | hCoV-19/SouthKorea/KDCA5957/2021 | South Korea | 2021-06-22 |
| EPI_ISL_3026147 | hCoV-19/SouthKorea/KDCA5995/2021 | South Korea | 2021-06-22 |
| EPI_ISL_2967439 | hCoV-19/SouthKorea/KDCA5725/2021 | South Korea | 2021-06-24 |
| EPI_ISL_3026291 | hCoV-19/SouthKorea/KDCA6178/2021 | South Korea | 2021-06-24 |
| EPI_ISL_3026215 | hCoV-19/SouthKorea/KDCA6072/2021 | South Korea | 2021-06-25 |
| EPI_ISL_3026290 | hCoV-19/SouthKorea/KDCA6177/2021 | South Korea | 2021-06-25 |
| EPI_ISL_3026306 | hCoV-19/SouthKorea/KDCA6194/2021 | South Korea | 2021-06-25 |
| EPI_ISL_2967502 | hCoV-19/SouthKorea/KDCA5789/2021 | South Korea | 2021-06-29 |
| EPI_ISL_2967542 | hCoV-19/SouthKorea/KDCA5832/2021 | South Korea | 2021-07-01 |
| EPI_ISL_2967543 | hCoV-19/SouthKorea/KDCA5833/2021 | South Korea | 2021-07-01 |
| EPI_ISL_3026478 | hCoV-19/SouthKorea/KDCA6435/2021 | South Korea | 2021-07-01 |
| EPI_ISL_3026451 | hCoV-19/SouthKorea/KDCA6388/2021 | South Korea | 2021-07-02 |
| EPI_ISL_3026529 | hCoV-19/SouthKorea/KDCA6509/2021 | South Korea | 2021-07-02 |
| EPI_ISL_3026537 | hCoV-19/SouthKorea/KDCA6518/2021 | South Korea | 2021-07-03 |
| EPI_ISL_3026554 | hCoV-19/SouthKorea/KDCA6536/2021 | South Korea | 2021-07-03 |
| EPI_ISL_3026547 | hCoV-19/SouthKorea/KDCA6528/2021 | South Korea | 2021-07-04 |
| EPI_ISL_2967577 | hCoV-19/SouthKorea/KDCA5868/2021 | South Korea | 2021-07-06 |
| EPI_ISL_3026652 | hCoV-19/SouthKorea/KDCA6646/2021 | South Korea | 2021-07-06 |
| EPI_ISL_3026765 | hCoV-19/SouthKorea/KDCA6777/2021 | South Korea | 2021-07-07 |
| EPI_ISL_3026791 | hCoV-19/SouthKorea/KDCA6811/2021 | South Korea | 2021-07-08 |
| EPI_ISL_3026819 | hCoV-19/SouthKorea/KDCA6840/2021 | South Korea | 2021-07-09 |
| EPI_ISL_3026844 | hCoV-19/SouthKorea/KDCA6875/2021 | South Korea | 2021-07-10 |
| EPI_ISL_3368931 | hCoV-19/SouthKorea/KDCA6920/2021 | South Korea | 2021-07-11 |
| EPI_ISL_3369219 | hCoV-19/SouthKorea/KDCA6949/2021 | South Korea | 2021-07-12 |
| EPI_ISL_3368945 | hCoV-19/SouthKorea/KDCA6975/2021 | South Korea | 2021-07-13 |
| EPI_ISL_3674097 | hCoV-19/SouthKorea/KDCA9105/2021 | South Korea | 2021-07-13 |

| EPI_ISL_3674130 | hCoV-19/SouthKorea/KDCA9117/2021 | South Korea | 2021-07-13 |
| --- | --- | --- | --- |
| EPI_ISL_3369112 | hCoV-19/SouthKorea/KDCA7126/2021 | South Korea | 2021-07-15 |
| EPI_ISL_3674192 | hCoV-19/SouthKorea/KDCA9138/2021 | South Korea | 2021-07-16 |
| EPI_ISL_3674298 | hCoV-19/SouthKorea/KDCA9165/2021 | South Korea | 2021-07-16 |
| EPI_ISL_3369096 | hCoV-19/SouthKorea/KDCA7307/2021 | South Korea | 2021-07-18 |
| EPI_ISL_3369251 | hCoV-19/SouthKorea/KDCA7297/2021 | South Korea | 2021-07-18 |
| EPI_ISL_3452119 | hCoV-19/SouthKorea/KDCA7763/2021 | South Korea | 2021-07-18 |
| EPI_ISL_3451923 | hCoV-19/SouthKorea/KDCA7457/2021 | South Korea | 2021-07-20 |
| EPI_ISL_3452111 | hCoV-19/SouthKorea/KDCA7758/2021 | South Korea | 2021-07-22 |
| EPI_ISL_3545008 | hCoV-19/SouthKorea/KDCA8702/2021 | South Korea | 2021-07-23 |
| EPI_ISL_3545074 | hCoV-19/SouthKorea/KDCA8793/2021 | South Korea | 2021-07-23 |
| EPI_ISL_3674368 | hCoV-19/SouthKorea/KDCA9196/2021 | South Korea | 2021-07-23 |
| EPI_ISL_3452180 | hCoV-19/SouthKorea/KDCA7807/2021 | South Korea | 2021-07-24 |
| EPI_ISL_3452237 | hCoV-19/SouthKorea/KDCA7852/2021 | South Korea | 2021-07-24 |
| EPI_ISL_3452263 | hCoV-19/SouthKorea/KDCA7878/2021 | South Korea | 2021-07-24 |
| EPI_ISL_3772635 | hCoV-19/SouthKorea/KDCA9557/2021 | South Korea | 2021-07-24 |
| EPI_ISL_3545117 | hCoV-19/SouthKorea/KDCA8854/2021 | South Korea | 2021-07-26 |
| EPI_ISL_3545123 | hCoV-19/SouthKorea/KDCA8868/2021 | South Korea | 2021-07-26 |
| EPI_ISL_3452291 | hCoV-19/SouthKorea/KDCA7904/2021 | South Korea | 2021-07-27 |
| EPI_ISL_3452389 | hCoV-19/SouthKorea/KDCA8003/2021 | South Korea | 2021-07-28 |
| EPI_ISL_3545155 | hCoV-19/SouthKorea/KDCA8915/2021 | South Korea | 2021-07-29 |
| EPI_ISL_3545189 | hCoV-19/SouthKorea/KDCA8974/2021 | South Korea | 2021-07-29 |
| EPI_ISL_3545166 | hCoV-19/SouthKorea/KDCA8941/2021 | South Korea | 2021-07-30 |
| EPI_ISL_3772722 | hCoV-19/SouthKorea/KDCA9648/2021 | South Korea | 2021-08-02 |
| EPI_ISL_3544787 | hCoV-19/SouthKorea/KDCA8354/2021 | South Korea | 2021-08-03 |
| EPI_ISL_3772919 | hCoV-19/SouthKorea/KDCA9632/2021 | South Korea | 2021-08-04 |
| EPI_ISL_3772923 | hCoV-19/SouthKorea/KDCA9414/2021 | South Korea | 2021-08-05 |
| EPI_ISL_3772927 | hCoV-19/SouthKorea/KDCA9424/2021 | South Korea | 2021-08-05 |
| EPI_ISL_3772939 | hCoV-19/SouthKorea/KDCA9425/2021 | South Korea | 2021-08-06 |
| EPI_ISL_3772966 | hCoV-19/SouthKorea/KDCA9439/2021 | South Korea | 2021-08-07 |
| EPI_ISL_3772899 | hCoV-19/SouthKorea/KDCA9514/2021 | South Korea | 2021-08-08 |
| EPI_ISL_3773012 | hCoV-19/SouthKorea/KDCA9462/2021 | South Korea | 2021-08-08 |
| EPI_ISL_3869621 | hCoV-19/SouthKorea/KDCA9855/2021 | South Korea | 2021-08-11 |
| EPI_ISL_3869627 | hCoV-19/SouthKorea/KDCA9862/2021 | South Korea | 2021-08-11 |
| EPI_ISL_3869680 | hCoV-19/SouthKorea/KDCA9926/2021 | South Korea | 2021-08-11 |
| EPI_ISL_3869741 | hCoV-19/SouthKorea/KDCA10039/2021 | South Korea | 2021-08-12 |
| EPI_ISL_3869714 | hCoV-19/SouthKorea/KDCA10001/2021 | South Korea | 2021-08-13 |
| EPI_ISL_3869784 | hCoV-19/SouthKorea/KDCA10103/2021 | South Korea | 2021-08-15 |
| EPI_ISL_3869823 | hCoV-19/SouthKorea/KDCA10154/2021 | South Korea | 2021-08-17 |
| EPI_ISL_3869895 | hCoV-19/SouthKorea/KDCA10256/2021 | South Korea | 2021-08-19 |
| EPI_ISL_4204052 | hCoV-19/SouthKorea/KDCA10778/2021 | South Korea | 2021-08-20 |
| EPI_ISL_3870023 | hCoV-19/SouthKorea/KDCA10463/2021 | South Korea | 2021-08-23 |
| EPI_ISL_4203836 | hCoV-19/SouthKorea/KDCA10595/2021 | South Korea | 2021-08-24 |
| EPI_ISL_4204082 | hCoV-19/SouthKorea/KDCA10801/2021 | South Korea | 2021-08-26 |
| EPI_ISL_4204126 | hCoV-19/SouthKorea/KDCA10863/2021 | South Korea | 2021-08-27 |
| EPI_ISL_4204322 | hCoV-19/SouthKorea/KDCA11141/2021 | South Korea | 2021-08-31 |
| EPI_ISL_4204340 | hCoV-19/SouthKorea/KDCA11175/2021 | South Korea | 2021-09-01 |
| EPI_ISL_4204401 | hCoV-19/SouthKorea/KDCA11259/2021 | South Korea | 2021-09-01 |

Table S3. Clinical characteristics of patients analyzed in this study

|  | **Patient 1** | **Patient 2** |
| --- | --- | --- |
| Sex | Male | Male |
| Age | 20 | 25 |
| Coexisting disorder | relapsed ALL | relapsed AML, GVHD, Evans syndrome |
| Immunosuppression | blinatumomab, inotuzumab ozogamicin, FLAG-IDA | prednisone, MMF |
| Exposure | 28-Aug-20 | 13-Nov-20 |
| Diagnosis | 02-Sep-20 | 20-Nov-20 |
| Admission | 18-Jun-20 | 20-Nov-20 |
| rRT-PCR  Ct (collection date) |  |  |
| NP swab | 14.88/17.03 (day 1), 17.88/19.37 (day 22), 16.14/17.3 (day 40),  16.41/17.06 (day 53) | 12.22/11.55 (day 0), 16.51/15.86 (day 12), 16.67/16.92 (day 19),  20.05/19.24 (day 29), 14.76/14.47 (day 38), 21.55/20.44 (day 52),  25.91/25.23 (day 59), 23.96/23.75 (day 63), 25.23/24.65 (day 67) |
| throat swab |  | 27.43/27.42 (day 19) |
| sputum | 12.92/14.97 (day 1), 16.6/18.32 (day 15), 17.03/18.32 (day 22),  22.07/23.46 (day 53), 20.12/21.26 (day 71), 26.21/26.04 (day 156) |  |
| saliva |  | 27.93/28.27 (day12), 21.15/21.15 (day 19), 24.05/24.09 (day 54) |
| stool |  | 24.83/25.17 (day12), 20.64/21.83 (day 19) |
| urine |  | 30.19/30.62 (day 19) |
| SARS-CoV-2 shedding (days) | 250 | 73 |
| Outcome | Deceased | Recovered |
| Symptom | fever | nasal congestion, fever* |
| Fever** (℃) | 38.4 | 39.5 |
| Radiologic findings | suggestive of bronchopneumonia | no pneumonic infiltration |

*considered to be unlikely to be a COVID-19 related symptom **The highest temperature during hospitalization period rRT-PCR, real-time reverse transcription-PCR

Table S4. Statistics of whole genome sequencing performed in this study.

| **WGS_ID** | **Patient** | **Sampling time** | **Sample type** | **Reads** | **Read count** | **Percentage of reads** | **Number of bases** | **Percentage of bases** | **Average depth (x)** |
| --- | --- | --- | --- | --- | --- | --- | --- | --- | --- |
| 1-S1 | Patient 1 | D1 | Sputum | Mapped reads | 336,131 | 99.68 | 49,886,922 | 99.74 | 1,675 |
|  |  |  |  | Not mapped reads | 1,085 | 0.32 | 130,827 | 0.26 |  |
|  |  |  |  | Total reads | 337,216 | 100.00 | 50,017,749 | 100.00 |  |
| 1-S2 |  | D1 | NP swab | Mapped reads | 470,420 | 99.67 | 67,644,758 | 99.76 | 2,263 |
|  |  |  |  | Not mapped reads | 1,580 | 0.33 | 163,732 | 0.24 |  |
|  |  |  |  | Total reads | 472,000 | 100.00 | 67,808,490 | 100.00 |  |
| 1-S3 |  | D15 | Sputum | Mapped reads | 328,722 | 97.93 | 48,810,124 | 98.04 | 1,641 |
|  |  |  |  | Not mapped reads | 6,956 | 2.07 | 977,046 | 1.96 |  |
|  |  |  |  | Total reads | 335,678 | 100.00 | 49,787,170 | 100.00 |  |
| 1-S5 |  | D22 | Sputum | Mapped reads | 305,042 | 96.09 | 45,326,427 | 96.23 | 1,530 |
|  |  |  |  | Not mapped reads | 12,400 | 3.91 | 1,774,442 | 3.77 |  |
|  |  |  |  | Total reads | 317,442 | 100.00 | 47,100,869 | 100.00 |  |
| 1-S6 |  | D22 | NP swab | Mapped reads | 357,103 | 99.62 | 52,935,834 | 99.68 | 1,781 |
|  |  |  |  | Not mapped reads | 1,377 | 0.38 | 169,145 | 0.32 |  |
|  |  |  |  | Total reads | 358,480 | 100.00 | 53,104,979 | 100.00 |  |
| 1-S7 |  | D40 | NP swab | Mapped reads | 312,785 | 99.58 | 46,294,221 | 99.67 | 1,554 |
|  |  |  |  | Not mapped reads | 1,319 | 0.42 | 152,460 | 0.33 |  |
|  |  |  |  | Total reads | 314,104 | 100.00 | 46,446,681 | 100.00 |  |
| 1-S8 |  | D53 | Sputum | Mapped reads | 213,674 | 82.03 | 31,652,815 | 82.41 | 1,070 |
|  |  |  |  | Not mapped reads | 46,802 | 17.97 | 6,757,975 | 17.59 |  |
|  |  |  |  | Total reads | 260,476 | 100.00 | 38,410,790 | 100.00 |  |
| 1-S9 |  | D53 | NP swab | Mapped reads | 316,350 | 97.07 | 47,099,874 | 97.17 | 1,586 |
|  |  |  |  | Not mapped reads | 9,546 | 2.93 | 1,371,158 | 2.83 |  |
|  |  |  |  | Total reads | 325,896 | 100.00 | 48,471,032 | 100.00 |  |
| 1-S12 |  | D71 | Sputum | Mapped reads | 125,060 | 39.36 | 18,574,863 | 39.66 | 626 |
|  |  |  |  | Not mapped reads | 192,702 | 60.64 | 28,258,892 | 60.34 |  |
|  |  |  |  | Total reads | 317,762 | 100.00 | 46,833,755 | 100.00 |  |
| 1-S17 |  | D156 | Sputum | Mapped reads | 269,279 | 72.18 | 39,930,851 | 72.44 | 1,347 |
|  |  |  |  | Not mapped reads | 103,763 | 27.82 | 15,190,051 | 27.56 |  |
|  |  |  |  | Total reads | 373,042 | 100.00 | 55,120,902 | 100.00 |  |
| 2-S1 | Patient 2 | D0 | NP swab | Mapped reads | 4,890,622 | 99.85 | 727,802,374 | 99.85 | 24,543 |
|  |  |  |  | Not mapped reads | 7,346 | 0.15 | 1,085,725 | 0.15 |  |
|  |  |  |  | Total reads | 4,897,968 | 100.00 | 728,888,099 | 100.00 |  |
| 2-S2 |  | D12 | NP swab | Mapped reads | 5,139,932 | 99.08 | 762,513,854 | 99.09 | 25,705 |

|  |  |  |  | Not mapped reads | 47,788 | 0.92 | 6,976,862 | 0.91 |  |
| --- | --- | --- | --- | --- | --- | --- | --- | --- | --- |
|  |  |  |  | Total reads | 5,187,720 | 100.00 | 769,490,716 | 100.00 |  |
| 2-R1 |  | D12 | saliva | Mapped reads | 401,660 | 97.68 | 59,281,421 | 97.94 | 1,990 |
|  |  |  |  | Not mapped reads | 9,528 | 2.32 | 1,249,913 | 2.06 |  |
|  |  |  |  | Total reads | 411,188 | 100.00 | 60,531,334 | 100.00 |  |
| 2-S4 |  | D12 | stool | Mapped reads | 5,241,934 | 94.29 | 779,161,714 | 94.35 | 26,279 |
|  |  |  |  | Not mapped reads | 317,688 | 5.71 | 46,668,714 | 5.65 |  |
|  |  |  |  | Total reads | 5,559,622 | 100.00 | 825,830,428 | 100.00 |  |
| 2-S6 |  | D19 | NP swab | Mapped reads | 4,968,617 | 99.84 | 738,927,656 | 99.84 | 24,855 |
|  |  |  |  | Not mapped reads | 7,833 | 0.16 | 1,150,793 | 0.16 |  |
|  |  |  |  | Total reads | 4,976,450 | 100.00 | 740,078,449 | 100.00 |  |
| 2-R4 |  | D19 | throat swab | Mapped reads | 424,120 | 99.49 | 62,381,701 | 99.66 | 2,097 |
|  |  |  |  | Not mapped reads | 2,164 | 0.51 | 214,858 | 0.34 |  |
|  |  |  |  | Total reads | 426,284 | 100.00 | 62,596,559 | 100.00 |  |
| 2-S7 |  | D19 | saliva | Mapped reads | 5,316,814 | 91.16 | 790,675,514 | 91.32 | 26,588 |
|  |  |  |  | Not mapped reads | 515,292 | 8.84 | 75,150,002 | 8.68 |  |
|  |  |  |  | Total reads | 5,832,106 | 100.00 | 865,825,516 | 100.00 |  |
| 2-S8 |  | D19 | stool | Mapped reads | 5,182,141 | 98.17 | 767,522,192 | 98.21 | 25,811 |
|  |  |  |  | Not mapped reads | 96,581 | 1.83 | 13,954,453 | 1.79 |  |
|  |  |  |  | Total reads | 5,278,722 | 100.00 | 781,476,645 | 100.00 |  |
| 2-R2 |  | D19 | urine | Mapped reads | 473,896 | 98.29 | 69,301,569 | 98.56 | 2,322 |
|  |  |  |  | Not mapped reads | 8,238 | 1.71 | 1,010,841 | 1.44 |  |
|  |  |  |  | Total reads | 482,134 | 100.00 | 70,312,410 | 100.00 |  |
| 2-S10 |  | D29 | NP swab | Mapped reads | 5,310,140 | 98.24 | 787,573,453 | 98.28 | 26,497 |
|  |  |  |  | Not mapped reads | 95,264 | 1.76 | 13,808,675 | 1.72 |  |
|  |  |  |  | Total reads | 5,405,404 | 100.00 | 801,382,128 | 100.00 |  |
| 2-S11 |  | D38 | NP swab | Mapped reads | 6,654,788 | 99.84 | 991,426,501 | 99.84 | 33,378 |
|  |  |  |  | Not mapped reads | 10,704 | 0.16 | 1,547,700 | 0.16 |  |
|  |  |  |  | Total reads | 6,665,492 | 100.00 | 992,974,201 | 100.00 |  |
| 2-S12 |  | D52 | NP swab | Mapped reads | 5,089,593 | 97.94 | 750,719,670 | 98.05 | 25,298 |
|  |  |  |  | Not mapped reads | 107,163 | 2.06 | 14,893,452 | 1.95 |  |
|  |  |  |  | Total reads | 5,196,756 | 100.00 | 765,613,122 | 100.00 |  |
| 2-R8 |  | D52 | saliva | Mapped reads | 369,119 | 76.61 | 54,332,117 | 77.13 | 1,819 |
|  |  |  |  | Not mapped reads | 112,675 | 23.39 | 16,109,842 | 22.87 |  |
|  |  |  |  | Total reads | 481,794 | 100.00 | 70,441,959 | 100.00 |  |
| 2-R10 |  | D59 | NP swab | Mapped reads | 496,135 | 83.43 | 73,208,602 | 83.98 | 2,449 |
|  |  |  |  | Not mapped reads | 98,571 | 16.57 | 13,964,724 | 16.02 |  |
|  |  |  |  | Total reads | 594,706 | 100.00 | 87,173,326 | 100.00 |  |

| 2-R11 |  | D63 | NP swab | Mapped reads | 464,553 | 92.88 | 68,439,009 | 93.30 | 2,292 |
| --- | --- | --- | --- | --- | --- | --- | --- | --- | --- |
|  |  |  |  | Not mapped reads | 35,593 | 7.12 | 4,912,142 | 6.70 |  |
|  |  |  |  | Total reads | 500,146 | 100.00 | 73,351,151 | 100.00 |  |
| 2-R12 |  | D67 | NP swab | Mapped reads | 271,206 | 58.24 | 39,939,743 | 59.20 | 1,327 |
|  |  |  |  | Not mapped reads | 194,432 | 41.76 | 27,525,764 | 40.80 |  |
|  |  |  |  | Total reads | 465,638 | 100.00 | 67,465,507 | 100.00 |  |
| 2-R3 |  | D52 | NP swab (mother) | Mapped reads | 419,561 | 93.78 | 61,654,164 | 94.21 | 2,072 |
|  |  |  |  | Not mapped reads | 27,811 | 6.22 | 3,786,216 | 5.79 |  |
|  |  |  |  | Total reads | 447,372 | 100.00 | 65,440,380 | 100.00 |  |

Table S5. SARS-CoV-2 variant and allele frequency changes in clinical samples.

Table S5 is uploaded as a separate file, due to its size.

Table S6. Transmission bottle size estimated using a beta-binomial model.

| **intra-household pair** | **Bottleneck size** | **95% Confidence interval (lower)** | **95% Confidence interval (upper)** |
| --- | --- | --- | --- |
| Patient 2 - mother | 3 | 2 | 5 |

References

1. GISAID (Global Initiative on Sharing All Influenza Data) home page [(https://www.gisaid.org/](https://www.gisaid.org/)).
